# Supplementary material for: Production of zosteric acid and other sulfated phenolic biochemicals in microbial cell factories
Source: Nat Commun. 2019 Sep 6;10:4071. doi: 10.1038/s41467-019-12022-x (PMC6731281; doi:10.1038/s41467-019-12022-x)
Supplement: Supplementary file 1 — Supplementary Information [file 41467_2019_12022_MOESM1_ESM.pdf]

Supplementary Materials

Production of zosteric acid and other sulfated phenolic biochemicals in microbial cell factories

Jendresen *et al*

SUPPLEMENTARY FIGURES

Supplementary Fig. 1 - Induction conditions for sulfotransferases

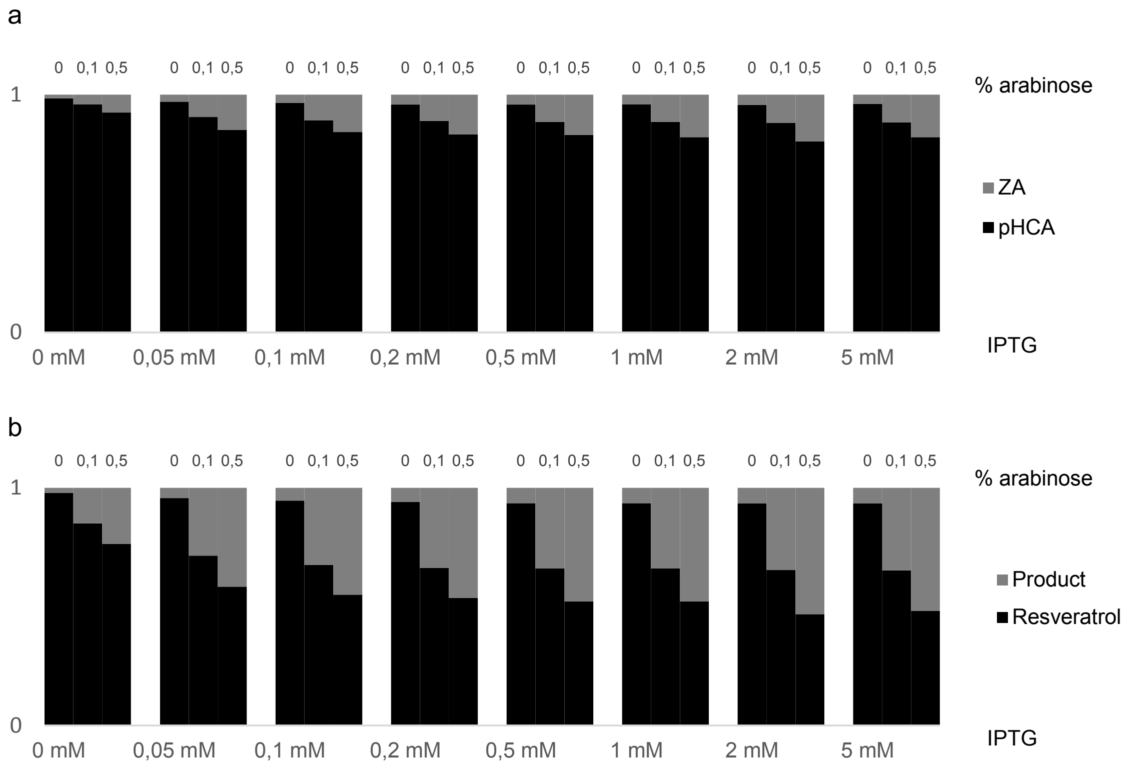

Supplementary Figure 1. CBJ1091 was grown in 96-well deep-well plates containing M9 with 0.2% glucose , 100 µg/mL ampicillin, 0.5 mM *p*-coumaric acid or 0.2 mM resveratrol as substrates, and dilutions of ITPG and rhamnose as indicated. After 24 h of growth at 37°C in an orbital shaker (300 rpm), the supernatants were isolated and examined by HPLC. The conversion of *p*-coumaric acid into zosteric acid by CBJ1091 (a) was quantified relative to standards of *p*-coumaric acid and zosteric acid. The results were similar for the conversion of resveratrol (b), where the peak areas were quantified.

Supplementary Fig. 2 - Product formation

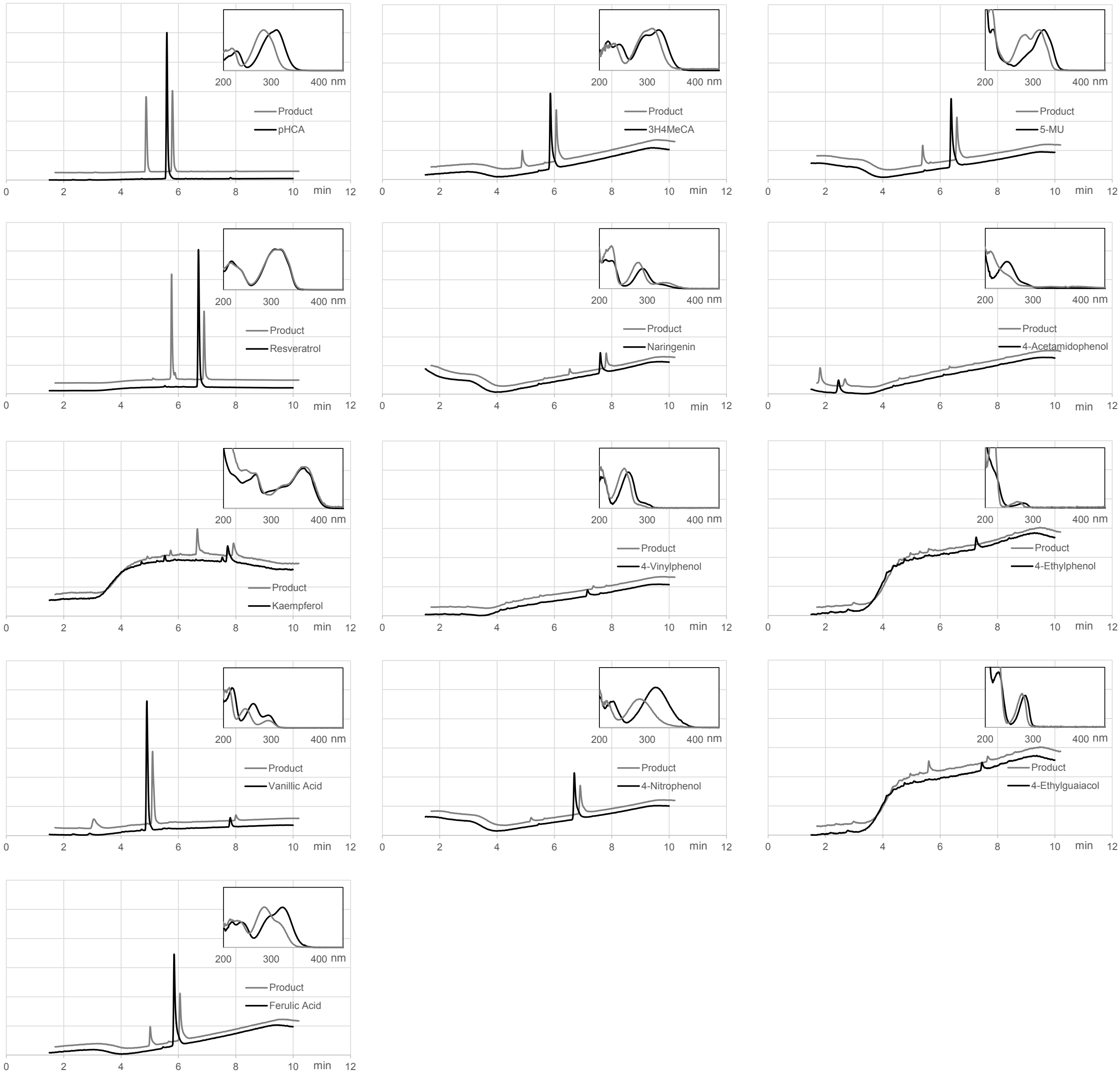

Supplementary Figure 2. Disappearance of 13 phenolic compounds and appearance of new compounds in culture media supernatant for strains expressing a sulfotransferase. UV-Vis chromotograms are shown with UV-Vis spectra for the original peak and the new peaks in the inserts. The conversion does not happen for the control strain (black lines). For the different compounds a specific sulfotransferase-expressing strain was used (grey lines). Here: CBJ1091 (*p*-coumaric acid and 3-hydroxy-4-methoxycinnamic acid), CBJ1093 (resveratrol), CBJ1143 (4-nitrophenol), CBJ1151 (kaempferol, vanillic acid, ferulic acid, 4-vinylphenol, 4-methylumbelliferone, 4-acetamidophenol, 4-ethylphenol, 4-ethylguaiacol) and CBJ1162 (naringenin). Abbreviations: 4-MU (4-methylumbelliferone), 3H4MeCA (3-hydroxy-4-methoxycinnamic acid).

Supplementary Fig. 3 - Inhibition of growth by *p*-coumaric acid

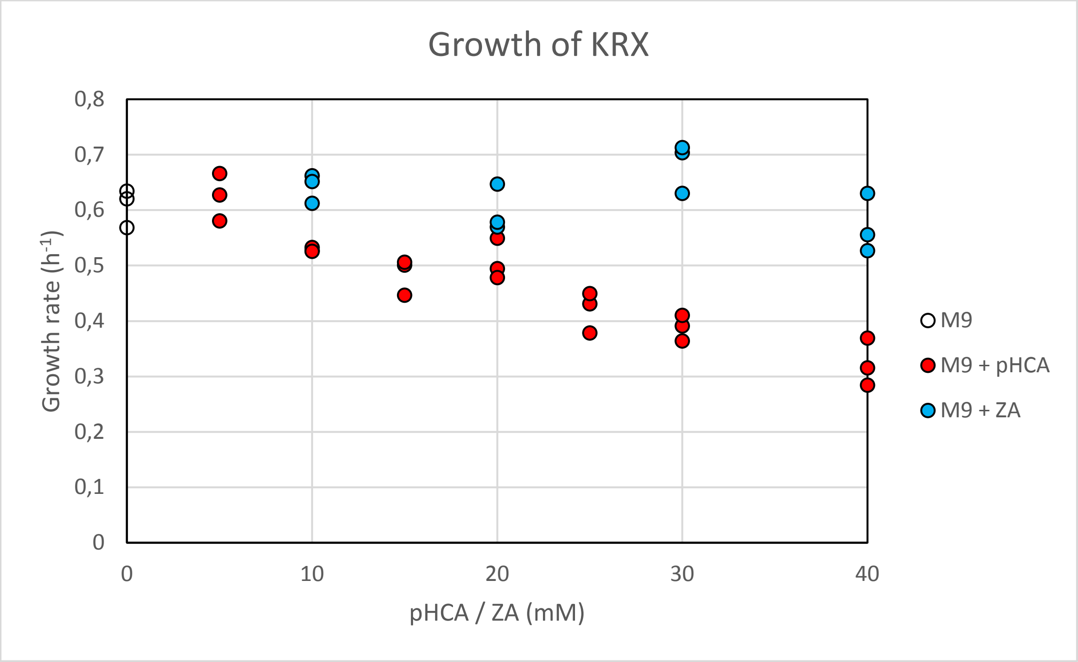

Supplementary Fig 3: Growth rate of *E. coli* in concentrations of *p*-coumaric acid and zosteric acid. *E. coli* was grown in the presence of the organic acids in pH-adjusted M9 minimal media with glucose at 37 °C with aeration. Three replicates per condition.

Supplementary Fig. 4 – Product stability

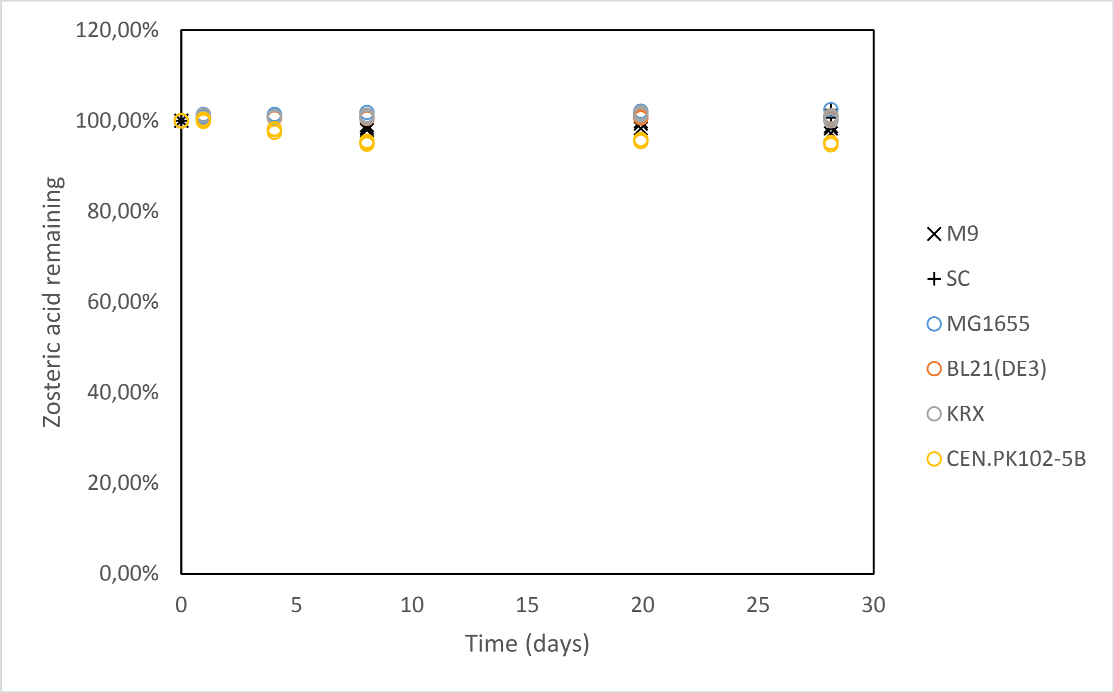

Supplementary Fig. 4: Stability of zosteric acid. M9 media and SC media were supplemented with zosteric acid to 2 mM and stored at 37°C and 30°C, respectively. Aliquots of M9 media were inoculated by 1000-fold dilution of *E. coli* cultures (either KRX, BL21(DE3), MG1655) in triplicates, and placed shaking at 37°C. Similarly SC was inoculated by *S. cerevisiae* CEN.PK102-5B in triplicates and placed shaking at 30°C. Samples were withdrawn during four weeks for quantification of zosteric acid.

Supplementary Fig. 5 – Effect of IPTG induction on growth rates

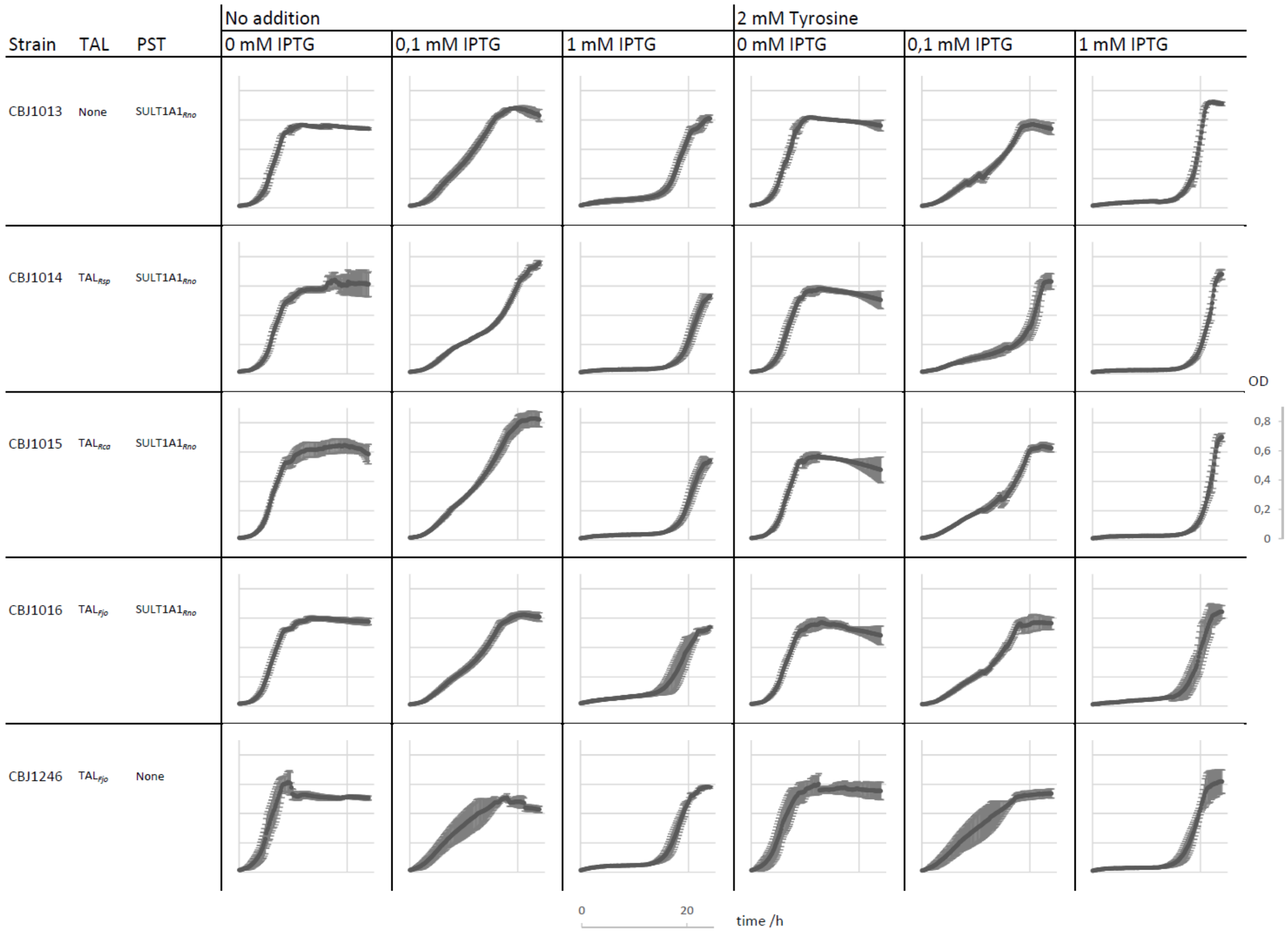

Supplementary Fig. 5: Effect of IPTG induction on growth rates. The same cultures as were used in table 1 were replicated (200 µL) to microtiter plates and the optical density was followed while the cultures are grown at 37°C with agitation. Averages of triplicate cultures are shown with standard deviations as error bars.

SUPPLEMENTARY TABLES

Supplementary Table 1: Production of zosteric acid in yeast

			No addition		2 mM Tyr		2 mM pHCA	5 mM pHCA
**Strain**	**PST**	**TAL**	**pHCA**	**ZA**	**pHCA**	**ZA**	**ZA**	**ZA**
CBJ981	None	None	0 ± 0	0 ± 0	0 ± 0	0 ± 0	0 ± 0	0 ± 0
CBJ973	None	$TAL_{Fjo, Sc}$	125 ± 5	0 ± 0	311 ± 9	0 ± 0	0 ± 0	0 ± 0
CBJ974	$SULT1A1_{Rno}$	None	0 ± 0	0 ± 0	0 ± 0	0 ± 0	33 ± 9	32 ± 7
CBJ975	$SULT1A1_{Rno, Ec}$	None	0 ± 0	0 ± 0	0 ± 0	0 ± 0	36 ± 6	34 ± 8
CBJ976	$SULT1A1_{Rno, Ec}$	TAL_{Ses}	10 ± 0	2 ± 0	11 ± 0	1 ± 0	32 ± 1	27 ± 1
CBJ978	$SULT1A1_{Rno, Ec}$	TAL_{Fjo}	56 ± 1	13 ± 0	111 ± 3	7 ± 0	29 ± 1	24 ± 3
CBJ980	$SULT1A1_{Rno, Ec}$	$TAL_{Fjo, Sc}$	89 ± 3	11 ± 0	236 ± 8	9 ± 0	17 ± 2	13 ± 1

Supplementary Table 1: Production of zosteric acid from glucose in minimal medium in recombinant yeast. $SULT1A1_{Rno}$ was expressed from an episomal 2µ-based plasmid in combination with selected tyrosine ammonia-lyases in *S. cerevisiae*, resulting in production of *p*-coumaric acid and zosteric acid. Strains were grown in minimal medium with glucose as a carbon source and addition of tyrosine or *p*-coumaric acid as indicated. Titers (µM) are shown with standard deviations (n = 3).
