# Supplementary material for: Production of zosteric acid and other sulfated phenolic biochemicals in microbial cell factories
Source: Nat Commun. 2019 Sep 6;10:4071. doi: 10.1038/s41467-019-12022-x (PMC6731281; doi:10.1038/s41467-019-12022-x)
Supplement: Supplementary file 4 — Description of Additional Supplementary Files [file 41467_2019_12022_MOESM4_ESM.pdf]

Description of Additional Supplementary Files

File Name: Supplementary Data 1
Description: Double-stranded DNA fragments encoding phenol sulfotransferases and 5’ (TAGAAATAATTTTGTTTAACTTTAAGAAGGAGATATACC) and 3’ (CAAGCTTGCGGCCGCATAATGCTTA) flanking regions.

File Name: Supplementary Data 2
Description: DNA oligonucleotides used in this study.

File Name: Supplementary Data 3
Description: DNA plasmids used in this study.

File Name: Supplementary Data 4
Description: Bacterial strains used in this study.
