# Supplementary material for: Production of zosteric acid and other sulfated phenolic biochemicals in microbial cell factories
Source: Nat Commun. 2019 Sep 6;10:4071. doi: 10.1038/s41467-019-12022-x (PMC6731281; doi:10.1038/s41467-019-12022-x)
Supplement: Supplementary file 5 — Supplementary Data 1 [file 41467_2019_12022_MOESM5_ESM.pdf]

Supplementary Data 1

Production of zosteric acid and other sulfated phenolic biochemicals in microbial cell factories

Jendresen *et al*

Supplementary Data 1: Double-stranded DNA fragments

Biobrick	Enzyme	DNA sequence
**CBJBB7**	SSU-1$_{Cel}$, *C. elegans* SSU-1	TAGAAATAATTTTGTTTAACTTTAAGAAGGAGATATACCATGACCCCGAAAACACCGAAA<br>ACCCCTAAACCGCCTCAGACACCGCGTCCGATGCTGACCGTTGGTAGCCCTCCGTGTACC<br>CCGTGTAGCCCGTTTGTTCTGAATGCAACCAGCTTTTGTTTTCGTAAAGGTCCGGCACGT<br>AGCGTTGTTTATCAGCCGAATGGTCATCCGAAACAGGTTGTTATTGATGGTGAAATTTGG<br>CCTCCGATCTTCAAACCGAAAAATGTTCGTACCGCAAAAAGCATGCAGTTTGGTGAAACC<br>GATGTTGTGATTGCAACCTATCCGAAATGTGGCACCACCTGGCTGCAGCATATCACCAGC<br>CAGCTGATTAAAGGTCATGATTATAAAGCCGGTAAAGGCAATGAACTGTGTGTTCAGAGC<br>CCGATGATTGAACGTATGGGTGCAGCATTTGCAGATAATATCAAAGGTCCGCGTGTTCTG<br>AAAACCCATTTTCATCATTATAACATCCCGAAATACCCGGATACCAAATATATCTATTGT<br>GTGCGCAATCCGAAAGATTGTCTGACCAGCTATTTCCATCATAATCGCAACTTCAAAATC<br>TATAATTGGGCAAATGGCACCTGGGATGTTTTTCTGGACCTGTTTGCAAGCGGTCAGCTG<br>GCATTTGGTGATTATTTTGAACATCTGCTGAGCTGGCTGCCGTGTCTGAAAGATGATAAT<br>GTTCTGTTCCTGAAATATGAAGATATGTTTCAGGATCTGGAAAACGCCGTGTATAAAATC<br>GGTCAGTTTCTGGGTGGTGAAGCAGCACATCGTGTTGAAAATCCGGAAATTCTGCGTGAA<br>ATTGTGGATAACAGCACCATTGATGCCATGAAAAAAGATCAGAAACGTTGGTTTCCGGAA<br>TCCCAGCTGCATAAAGTTGAATTCATTCGCAAAGGTGGTAGCCGTGATTGGAAAAACTAT<br>TTTACCCGTGAACAGAGCGATCGCATTGATAGCATTTTTGCAGCAAAATTTGCAGGTACA<br>CCGGCAGAACATTGGTGGAAATATGAGATGGCATGGGAAGAAAAACCGCTGAGCATTGAA<br>AATCTGTCTATGGAAGAAGAAGAGGAAGAACAGTCACAGAAACTGTTTGCACTGCCTCCG<br>CTGCCACCGCAGCGTCGTTTTAGCCGTACCAGCCTGCTGAGCGCAGGTTATGGTAGCGTT<br>TGGAGCCTGAGCAGCCAGAATGCAAATATGAGCGCAAGCAGCAGCGTTAATAAAGATCTG<br>AGCACATTTGCCGAGTAACAAGCTTGCGGCCGCATAATGCTTA
**CBJBB8**	SULT1ST1$_{Dre}$, *D. rerio* SULT1ST1	TAGAAATAATTTTGTTTAACTTTAAGAAGGAGATATACCATGGACATCCCGGATTTTAGC<br>AGCATTAGCAGCCGTCCGACCATTTTTGAATTTGAAGGTATTAGCATGATCAACCACTTT<br>ACCGAAAACTGGGAGAAAGTGAAAAACTTTCAGGCACGTCCGGATGATATTCTGATTGCA<br>ACCTATCCGAAAGCAGGCACCACCTGGGTTAGCTATATTCTGGATCTGCTGTATTTTGGT<br>GAAAACGCACCGGAAGAACATACCAGCCAGCCGATTTATATGCGTGTTCCGTTTCTGGAA<br>AGCTGCTTTAAAGTTATTGCAAGCGGCACCGAACTGGCAGATAATATGACCACCAGTCCG<br>CGTCTGATTAAAACCCATCTGCCGGTTCAGCTGATTCCGAAAAGTTTTTGGGAACAGAAT<br>AGCCGTGTTGTTTATGTTGCACGTAATGCCAAAGATAACGTGGTGAGCTATTTTCATTTT<br>GATCGCATGAATATCGTTGAACCGGATCCTGGTGATTGGAATACCTTTCTGCATCGTTTT<br>ATGGATGGCAAAAGCGTTTTTGGTCCGTGGTATGATCACGTTAATGGCTATTGGGAAAAA<br>AAACAGACCTATAGCAACCTGCTGTACCTGTTTTATGAAGATCTGGTTGAAGATACCGGT<br>CGTGAAGTTGATCGTCTGTGTAGCTTTCTGGGTCTGAGCACCAGCGTTAGCGATCGTGAA<br>AAAATCACCAAAGATGTTCAGTTCGATGCCATGAAACAGAACAAAATGACCAATTATAGC<br>ACCCTGCCGGTGATGGATTTCAAAATTTCACCGTTTATGCGCAAAGGCAAAGTTGGCGAT<br>TGGAAAAATCATTTTACCGTGGCACAGAACGAACAGTTTGATGAGGTGTACAAAGAAAAA<br>ATGAAAAACGCCACCGTGAAATTTCGCACCGAAATCTAACAAGCTTGCGGCCGCATAATG<br>CTTA
**CBJBB9**	SULT4A1$_{Dre}$, *D. rerio* SULT4A1	TAGAAATAATTTTGTTTAACTTTAAGAAGGAGATATACCATGGCCGAAAGCGAAGTTGAT<br>ACCCCGAGCACCCCGATTGAATATGAAAGCAAATATTTCGAACATCATGGTGTGCGTCTG<br>CCACCGTTTTGTCGTGGTAAAATGGATGAAATTGCCAATTTTAGCCTGCGCAGCAGCGAT<br>ATTTGGATTGTTACCTATCCGAAAAGCGGCACCAGCCTGCTGCAAGAAGTTGTTTATCTG

		GTTAGCCAGGGTGCAGATCCGGATGAAATCGGTCTGATGAATATTGATGAACAGCTGCCG<br>GTTCTGGAATATCCGCAGCCTGGTCTGGAAATTATTCAAGAACTGACCAGTCCGCGTCTG<br>ATTAAAAGCCATCTGCCGTATCGTTTTCTGCCGAGCGCAATGCATAATGGTGAAGGTAAA<br>GTGATTTATATGGCACGCAATCCGAAAGATCTGGTGGTTAGCTATTATCAGTTTCATCGT<br>AGCCTGCGTACCATGAGCTATCGTGGCACCTTTCAAGAATTTTGTCGTCGCTTTATGAAC<br>GACAAACTGGGTTATGGTAGCTGGTTTGAACATGTGCAAGAATTCTGGGAACATCGTATG<br>GATAGCAATGTGCTGTTTCTGAAATATGAAGATATGTATAAAGACCTGGGCACCCTGGTG<br>GAACAGCTGGCACGTTTTCTGGGTGTTAGCTGTGATAAAGCACAGCTGGAAAGCCTGGTT<br>GAAAGCAGCAACCAGCTGATTGAACAGTGTTGTAATAGCGAAGCACTGAGCATTTGCCGT<br>GGTCGTGTTGGTCTGTGGAAAGATGTTTTTACCGTTAGCATGAACGAGAAATTCGATGTG<br>ATTTACCGTCAGAAAATGGCGAAAAGCGATCTGACCTTTGATTTTATCCTGTAACAAGCT<br>TGCGGCCGCATAATGCTTA
**CBJBB10**	SULT6B1$_{Dre}$, *D. rerio* SULT6B1	TAGAAATAATTTTGTTTAACTTTAAGAAGGAGATATACCATGAGCCAGATGAAAAGCCGT<br>ATGGAAACCGCAGCAAAAATGAAAGATGAGGATAAACTGTATCGCCGTGATGGTATTCTG<br>TATAGCACCGTTCTGAGCCCTCCGGAAACCCTGGACAAACTGAAAGATCTGCAGGCACGT<br>GAAGATGATCTGATTCTGGTTGCATATCCGAAATGCGGTTTTAATTGGATGGTTGCAGTT<br>CTGCGCAAAATCATTAATGCAAGCACCGGCAAAGATGAAAAACCGCCTGAACGTCCGCCT<br>CTGGTTGAATTTCTGCCTCCGACCGTTCAAGAAGAAATGGCCCAGATGCCTCCGCCTCGT<br>CTGCTGGGCACCCATCTGCATCCGGATAATATGCCTGCAACCTTTTTTACCAAAAAACCG<br>AAAATCCTGGTGGTGTTTCGTAATCCGAAAGATACCCTGGTTAGCTATTATCACTTTATG<br>AACAAAAATCCGGTTCTGCCGAATGCAGAAAGCTGGGATAAATTCTTTAGCGATTTTATG<br>ACCGGTGATGTTAGCTGGGGTAGCTATTTTGATCATGCACTGGCATGGGAAAAACGTATT<br>GATGATCCGAATGTGATGATCGTGATGTATGAAGATCTGAAACAGAATCTGCCGGAAGGC<br>GTGAAAAAAATCAGCGAATTTTTCAGCCTGCCGCTGACCGATGAACAGGTTAGCAGCATT<br>GCAGGTCAGAGCACCTTTAGCGCAATGGTTGAAAATTCACAGAAAAGCCATGGTAACTTT<br>GGCAGCATCTTTTTTCGTAAAGGTGAAGTGGGTGATTGGAAAAACCATTTTAGCGAAGCA<br>CAGAGCAAACAAATGGATGAGCTGTATCATAGCAAACTGGCAGGTACAAAACTGGCTGCA<br>CGTATGAATTATGATCTGTATTGCCAGTAACAAGCTTGCGGCCGCATAATGCTTA
**CBJBB11**	SOT12$_{Ath}$, *A. thaliana* AtSOT12	TAGAAATAATTTTGTTTAACTTTAAGAAGGAGATATACCATGAGCAGCAGCAGCTCAGTT<br>CCGGCATATCTGGGTGATGAAGATCTGACCCAAGAAACCCGTGCACTGATTAGCAGCCTG<br>CCGAAAGAAAAAGGTTGGCTGGTTAGCGAAATCTATGAATTTCAGGGTCTGTGGCATACC<br>CAGGCAATTCTGCAGGGTATTCTGATTTGTCAGAAACGCTTTGAAGCCAAAGATAGCGAT<br>ATTATTCTGGTGACCAATCCGAAAAGCGGCACCACCTGGCTGAAAGCACTGGTTTTTGCA<br>CTGCTGAATCGTCATAAATTTCCGGTTAGCAGCAGCGGTAACCATCCGCTGCTGGTTACA<br>AATCCGCATCTGCTGGTTCCGTTTCTGGAAGGTGTTTATTATGAAAGTCCGGATTTTGAT<br>TTTTCGAGCCTGCCGTCACCGCGTCTGATGAATACCCATATTAGCCATCTGAGTCTGCCG<br>GAAAGCGTTAAAAGCAGCAGTTGTAAAATTGTGTATTGCTGCCGTAACCCGAAAGATATG<br>TTTGTTAGCCTGTGGCACTTTGGCAAAAAACTGGCACCGGAAGAAACCGCAGATTATCCG<br>ATTGAAAAAGCAGTTGAAGCCTTTTGCGAAGGCAAATTTATCGGTGGTCCGTTTTGGGAT<br>CATATCCTGGAATATTGGTATGCCAGCCGTGAAAATCCGAATAAAGTTCTGTTTGTGACC<br>TATGAAGAACTGAAAAAACAGACCGAGGTGGAAATGAAACGTATTGCAGAATTTCTGGAA<br>TGCGGCTTTATTGAAGAAGAAGAGGTTCGCGAAATTGTGAAACTGTGTAGCTTTGAAAGC<br>CTGAGCAATCTGGAAGTGAATAAAGAAGGTAAACTGCCGAACGGCATTGAAACCAAAACC<br>TTTTTTCGCAAAGGTGAAATTGGTGGTTGGCGTGATACCCTGAGCGAAAGCCTGGCAGAA<br>GAAATTGATCGTACCATCGAAGAAAAATTCAAAGGCAGCGGTCTGAAATTTAGCAGCTAA<br>CAAGCTTGCGGCCGCATAATGCTTA
**CBJBB12**	*Streptomyces* Cpz8	TAGAAATAATTTTGTTTAACTTTAAGAAGGAGATATACCATGAAAATCATTGGTGCAGGT<br>TTTGGTCGTACCGGCACCCTGAGCGTTAAAGCAGCACTGGAAACCCTGGGTCTGGGTCCG<br>TGTTATCATATGCTGACCACCTTTGAAGAACCGGGTCATCTGCGTCTGTGGAATGCAGTT<br>AGCCGTGGTGAACGTGTTGATTGGGCAGAAATTTTTGCACGTTATCGTAGCACCGTTGAT<br>TGGCCTGCATGTGATCATTGGGAAACACTGGCAAAAGAATATCCGGAAGCAAAAGTTCTG<br>CTGACCGTTCGTGATAGCGAACGTTGGTATGATAGCTTTCGTCAGACCCTGGCACCGCTG<br>TGGTCTGCAGAAAGCGCAGATCCGGAACTGGCAGAATATCTGGATCTGGTTCGTCATATT<br>ACCGCACATACCTTTGGTGGTCGTCTGGATGATCGTGCACATGCAATTGCCGTTTTTGAG<br>GAACATAATCGTCGTGTTCGTGCAAGCATTCCGAGCGAACGTCTGCTGGTTTTTGATGTT<br>CGTGAAGGTTGGGAACCGCTGTGTGCATTTTTTGGCCGTCCGGTTCCGCCTGATACCCCG<br>TTTCCGCATCTGAATGATCGCGCAGCATTTCAAGAACTGCTGAGCCGTCGTCTGGCACAT<br>CGTGGTGATAGCCGTTAACAAGCTTGCGGCCGCATAATGCTTA

**CBJBB13**	*Streptomyces* LipE	TAGAAATAATTTTGTTTAACTTTAAGAAGGAGATATACCATGAAAATCATTGGTGCAGGT<br>TTTGGTCGTACCGGCACCCTGAGCGTTAAAGCAGCACTGGAAACCCTGGGTCTGGGTCCG<br>TGTTATCATATGCTGACCGCATTTGAAGAACCGGGTCATCTGCGTCTGTGGAATGCAGCA<br>GGTCGTGGTGAACGTGTTGATTGGACCGAAATTTTTGCACGTTATGAAAGCACCGTTGAT<br>TGGCCTGGTTGTGATCATTGGGAAACACTGGCAAAAGAATATCCGGATGCCAAAATTCTG<br>CTGACCGTTCGTGATCCGGAAGGTTGGTATGATAGCTTTCTGCAGACCCTGGCACCGCTG<br>TGGTCAGCCGAAAGTGATGATCCTGAAGTTCGTGAATATCTGGAACTGGTTCGTCGTATT<br>AGCGCACGTACCTTTGATGGTCGTCTGGATGATCGTGCACATGCAATTGCCGTTTTTGAA<br>GATCATATTCGTCGTGTTCGTGCAGCAGTTCCGGCAGATCGTCTGCTGGTTTATGATGTT<br>CGTCAGGGTTGGGAACCGCTGTGTGCATTTTTTGGCCGTCCGGTTCCGCCTGATACCGAT<br>TTTCCGCATCTGAATGATCGCGCACGTTTTCAAGAACTGCTGAGCCGTCGTTTTGCACAG<br>CGTGGTGATCCGCGTTAACAAGCTTGCGGCCGCATAATGCTTA
**CBJBB14**	*S. punctatus* DAOM BR117 SPPG_07427	TAGAAATAATTTTGTTTAACTTTAAGAAGGAGATATACCATGCCGCTGGAAATTATTGGT<br>GCAGGTTTTGGTCGTACCGGCACCAAAAGTCTGCAGCTGGCACTGCAGCAGCTGGGTTTT<br>CCGTGTTATCATATGACCGAAGCAATTCAGCATCCGACCGATTTTGCAGTTTGGACCGAT<br>GCACATGATGGTAAACAGGTTGATCTGACCCAGATCTTCAACAAATATACCGCAACCCTG<br>GATTGGCCTGCATGTGATTTTTGGAAAGAACTGATGGAAGTTTACCCGAACGCAAAAGTT<br>ATTCTGAGCGTTCGTGATGCAGATACCTGGTATGAAAGCGTGCGTGAAACCGTGTATAAA<br>CTGAATATGCTGCCTCGTAGCATCATTCAGAATTATCCGGACATTGTGAACGTGAACGAA<br>TATTGTCAGCATAGCATTTGGGGTCTGGGTGGTAACTTTAAAGGTCAGTTTGAAAATCCG<br>GCAATCGCCAAAAAAATCTTTCGTGATCGTCTGGAAGAAGTGAAACGTAGCGTTCCGAGC<br>GAAAAACTGCTGGTTTTTGAAGTTAGCCAGGGTTGGGAACCGCTGTGTAAATTTCTGGAA<br>CGTCCGGTTCCGAATGAACCGTTTCCGCGTACCAATGAACGTGCACTGTTTCAGAGCATG<br>ATTCAGGATATTATGACCGGTCGTCCGATTGATAAACGTCCGAATTTTCCGGATCTGCGT<br>CAGAATATTGGTGAAGCAGGTAAAGTTGATGGTGTGAGCGAAAACTAACAAGCTTGCGGC<br>CGCATAATGCTTA
**CBJBB15**	*H. ochraceum* DSM 14365 Hoch_6098	TAGAAATAATTTTGTTTAACTTTAAGAAGGAGATATACCATGAGCATGGATATTATTGGT<br>GCAGGTATGGGTCGTACCGGCACCCTGAGCCTGAAACATGCACTGGAACGTCTGGGTTTT<br>GATCCGTGTTATCATATGACCACCCTGCTGGAACATCCGGAACATGCAAGCACCTGGGCA<br>GCAGCAGCACGTGGTGAACAGGTTGATTGGAATACCGCACTGGGTAGCTTTCGTGCAACC<br>GTTGATCATCCGGGTTGTAACTTCTATAAAGATCTGATGGCAAAATATCCGGAAGCCAAA<br>GTTATTCTGAGCGTTCGTGATCCGGGTAAATGGTATGATAGCGCACGTGAAACCATTTAT<br>CGTGCAAGCCGTGCAGCAATTGCAGCCGGTGCACGTGAAGGTGCACCGGAAATTATGCGT<br>GTTGCAAATAGCCTGGTTTGGGAAAAACAGTTTGATGGTCGTTTTGAAGATCGCGAATAT<br>GCCATTAGCGTTTTTGAACAGCATAACGAAGAAGTGAAACGTACCGTTCCGGCAGAACGT<br>CTGCTGGTTTTTGAAGCACGTCAGGGTTGGGAACCGCTGTGTGCATTTCTGGGTGTTGAT<br>GTGCCGGATGAACCGTATCCGCGTGTTAATAGCCGTGAAGAATTCAAAAAAATGATCGCC<br>GAAATGGAAGGCGAAGCCTAACAAGCTTGCGGCCGCATAATGCTTA
**CBJBB16**	*R. radiotolerans* RradSPS_0172	TAGAAATAATTTTGTTTAACTTTAAGAAGGAGATATACCATGGATGGTGAACTGCGTGTT<br>ATTGGTGCAGGTTTTGGTCGCACCGGTACAACCAGCCTGAAAGCAGCACTGGAACGTCTG<br>GGCTTTGGTCCGTGTTATCACATGACCGAAGTTCTGCGTCATCCGAGCCATGCAAGCTTT<br>TGGAATGAAGCACATGAACGTGCCGGTGATGTTGATTGGCGTGGTTTTTTTCGTGATAGC<br>GGTGATTATCGTAGCGCAGTGGATTGGCCTGTTTGTGATTTTTATCGTGTTCTGATGGAT<br>GAATATCCGCGTGCAAAAGTTATTCTGACCGTTCGTGATCCTGATCGTTGGTATCGTAGT<br>GCACGTGAAACCATTTATGCACTGGGTAATGCATGGGCAGGTCGTTTTGTTTTTGCAACC<br>GTTGGTCGTCTGGTTCCGAGCTTTGGTCGTATGATGCGTATGGCACATCGTCTGATTTGG<br>GCAGGCACCTTTGGTGGTCGTTTTACCGATGAAGAACATGCCAAACGTGTTTTTCTGGAA<br>CATGATCGTGAAGTTCGTCGTACCGTTCCGCCTGAACGTCTGCTGGTTTATGATGTTAAA<br>GAGGGTTGGGGTCCGCTGTGTAAATTTCTGGGTGTTGAAGTTCCGGATGAACCGTTTCCG<br>CATCTGAATGATGCAGCAACCTTTCGTCGCCTGCTGTGGCTGCAGCGTGCATATGCCGTT<br>CTGCTGCCTGCAAGCCTGCTGGCACTGCTGCTGCTGGCCCTGCGTCGTGCCCGTGATGGT<br>GGTTAACAAGCTTGCGGCCGCATAATGCTTA
**CBJBB17**	*Zostera marina* KMZ76263.1	TAGAAATAATTTTGTTTAACTTTAAGAAGGAGATATACCATGGCAGGTATTCTGGCACTG<br>GAAAAATGTTTTGGCAGCAAAAACGAGCAAGAGAAAGAAGAGGATTCCAAAATGTATAAA<br>CGCTATCGCGAAATTGTTAGCAGCCTGCCGAGCAATGATTATTGGGGTGATACCATGCGT<br>CTGTATAAAGGTTTTTGGCAGATGGGTTATCTGGTTCCGGGTATTATGGCATTTGAGGAT<br>AACTTTAAAGCACGCGAAACCGATATCATTCTGACCACACTGCCGAAAGCAGGCACCACC<br>TGGACCAAAGCACTGACCTTTGCAATTCTGACCCGTGATGTTAATCATCCGAGCAGCCCG<br>ACACATCCGCTGCTGTTTTTTAACCCGCATAGCTGTGTTCAGAATCTGGAATATCTGTAT

		ATGGGTCGCGAAAATACCATGCCGGATCTGGATATGCTGAATGAAAGTCCGCGTCTGTTT<br>GCAGGTCATATTCCGTATAGCCTGCTGCCTGCAAGCGTTCTGAAAAGCGGCACCAAAATC<br>ATTAACATTAGCCGTAATCGCAAAAGCACCTTTGTGAGCTTTTGGAAATTTGGCAATCTG<br>ATCAATCCGGATAAACTGCTGGATCTGGAAAAAAAGCGTTGATATTTTTGCAAGCGGCATT<br>AGCTTTTGTGGTCCGGAATGGAATTTTCAGGCAGAATTTACCAATGCAGCAAGCACCAAT<br>AGCAATCTGCTGCTGCTGAGCTATGAAGAAATGCTGGAAAAACCGGTGGAAAACGTGAAA<br>AAACTGGCCGAATTTATGGGTTGTGGTTTTACCGATGATGAAGAAAAACAGGGCATTGTG<br>GATGAAATTGTGAAACTGTGCAGCTTCGATAACCTGAAAAATCAGCAGGTCAATAAAAAC<br>GGCTCCAGCTATAACAGCAAAATCGACAACAAACACTTTTTCCGCAAAGGTGAAGTTCGT<br>GATTGGGCAAATTATCTGACACCGGAAATGATCAAAAAGCTGGAAACCGCAGGTAAAATC<br>AACGAAAGCGAATAACAAGCTTGCGGCCGCATAATGCTTA
**CBJBB18**	*Zostera marina* KMZ64288.1	TAGAAATAATTTTGTTTAACTTTAAGAAGGAGATATACCATGGATCCGAGCATGGATGAA<br>TGTGAAGTTTTTAGCGGTGTTGTGGATGATGATCAGCAAGAAACCATCAAAGAAAGCTTT<br>GCCAAATATCGTCAGCTGGTTGCCACCTTTCCGCGTTGTACCCGTCTGAGCAGCGAATTT<br>GCACCGCTGGAAATTACCTATTTTCAGCATAAAGAACACGGCTGGATTAACACCTTTCTG<br>ATTATGGTTAATTGTCTGGTGGCCCAGAAACATTTTGTTGGTCGTCCGACCGATGTGATT<br>ATTAGCAGCCTGCCGAAAAGCGGCACCATTTGGCTGAAAGATCTGGTTTGTAAAATTACC<br>GGTCGTGGTAATCCGGATCATAAAAATGATCTGCTGAGTCCGCATCAGAAAGTTCCGTTT<br>CTGGAACTGCAGGTTTATGTTAGCGAAGATCATGTGCTGGATATTGATAGCTTTCCGAGT<br>CCGCGTCTGCTGAGCACCCATATTCCGTATCCGAGCCTGCCTGCAAGCCTGATTAATAGC<br>GGTTGTCCGATTGTGTATATTTGGCGTGATCCGAAAGCAATCTTTGTTAGCGATTGGCAC<br>TTCTTCAACAAAATCATTCCGAGCGAAACCGGCACCAATGTGCCGTTTCTGACCATTGAT<br>GAAAAATTCGATAGCTTCTGTCAGGGCTATAGCATTAGCGGTCCGTATTGGGATCATGTT<br>TTAGGTTATTGGAACGCCAAAAAAAACGGTGCCAATATCCTGCTGATCAAATATGAAGAT<br>CTGATGGCAGATCCGGTTGTTCATCTGAAAACCCTGGCAGAATTTCTGAAATTTCCGTTT<br>ACCGAAGAGGAAGAGAAAGACAACGTTATCCAGGATATTATCACCACCTGTAGCTTTCAG<br>AAAGTGAAAGACAGCAAAATGTATGAAAGCGGTGTTACCAAACTGCTGCATGTGGATATT<br>CAGAATACCATGTTTCTGCGTGAAGGCAAAACCAATGATTGGGAAAATTATCTGACCCCG<br>AAAATGGCAGAACGTCTGGATCTGATTACCATTCAGCGTTTTGCAGATACCGATCTGATC<br>TAACAAGCTTGCGGCCGCATAATGCTTA
**CBJBB19**	*Zostera marina* KMZ76264.1	TAGAAATAATTTTGTTTAACTTTAAGAAGGAGATATACCATGATCGGCATTCATACCGGT<br>GATCAGATGGCAGGTATTCTGCCGGTTGAACGTTGTTTTGCACCGAAAAAACAAGAAGAA<br>ACCGAAGAGGATAGCCAGATGTATAAAAGCTATGCCGAAATTGTTAGCAGCCTGCCGTTT<br>GTTGATAGCTGGGGCACCAAACTGGTTCTGTATAATGGTCTGTGGGCAATTGATGAACTG<br>CTGCCTGGTATTATTGCATTTCGCGATCATTTTAAAGCCCGTGAAACCGATATTATTGTT<br>GCCACCTTTCCGAAAGCAGGCACCACCTGGACCAAAGCACTGGCATTTACCATTCTGACC<br>CGTGAAGTTAATCATCCGAGCAGCCCGACACATCCGCTGCTGGGTTTTAATCCGCATAGC<br>TGTGTTACCACACTGGAATATCTGTATATGGGTCGTGAAAATCTGATGCCGGATACCGAT<br>GTTCTGAATGAAAGTCCGCGTCTGTTTGCAACCCATGTTCCGTATAGCTTTCTGCCGAAA<br>AGCATTGTTGAAAGCGGTGCCAAAATCATTAATGTTAGCCGTGAACGTAAAAGCACCTTT<br>GTTAGCCAGTGGAAATTCTATGGTGATATTTGGGAAGATGGTGATAGCGATAGCCTGGAT<br>CTGGAAAAATATGTTGACCTGTTTACCAGCGGTATTAGCCTGTATGGTCCGGATTGGGTT<br>TATCGTGCAGAATATACCAATGCAAGCAGCACCAATAGCAATCTGCTGCTGCTGAGCTAT<br>GAAGAGATGATTGAAAAACCGGTGGAAAACGCCAAAAAAATGGCAGAATTTATGGGTTGC<br>GGTTTCACCGATGATGAAGTTAAACTGGGTATTGTGGATGAGATTGTTAAACTGTGCAGC<br>TTCGATAATCTGAAAAGCCAGCAGGTTAACAAAAATGGTCGTAGCTTTAGCATGATCGAC<br>AACAAACACTTTTTTCGCAAAGGTCAGGCAGGCGATTGGGTGAATCATCTGAGTCCGGAA<br>ATGGTTAGCAAACTGGAAAAAGTTGCCGGTAAAGATAATGGCCAGTAACAAGCTTGCGGC<br>CGCATAATGCTTA
**CBJBB20**	*Zostera marina* KMZ59959.1	TAGAAATAATTTTGTTTAACTTTAAGAAGGAGATATACCATGGCCGAATGCGAAGTTTTT<br>AGCGGTCTGGTTAGCACCGATGAAGATATTGTTGATGATCAGCAAGAAACCATCAAAGAG<br>AGCTTTGCAAAATATCGTCAGCTGGTTGCCACCTTTCCGCGTTGTACCTGTCTGAGCAGC<br>GAATGTGTTCCGCTGGAAACCGCATTTTTTCAGCATAAAGAACATGGTTGGATTAGCCGT<br>TTTCCGACCATTGTTAATTGTCTGGTTGCCCAGAAATACTTTGTTGGTCGTAGTACCGAT<br>GTTTTTGTTAGCAGCCTGCCGAAAAGCGGCACCGTTTGGCTGAAAGATCTGGTGTATAAA<br>ATCAAACGTCGTGGTGGTAACCAGGATCATAAAAATGATCTGCTGAGTCCGCATCAGAAA<br>GTTCCGTTTCTGGAACTGCAGGTTTATGCAAGCGAAGATCATGTTCTGGATATTGATAGC<br>CTGCCGTCACCGCGTCTGCTGAGCACCCATATTCCGTATCCGAGTCTGCCGACCAGCCTG<br>ATTGATAGCGGTTGTCCGATTGTGTATATTTGGCGTGATCCGAAAGCAGTTTTTGTGAGC

		GATTGGCACTTTTTCAACGAAATCATTCCGAGCGAACCGGGTAAAAATCATCCGCCTCTG<br>ACCATTAACGAAAAATTTGAATGCTTCTGCAACGGCTATAGCATCTTTGGTCCGTATTGG<br>GATCATGTGTTAGGTTATTGGAACGCCAAAAAAAACGGTGCGAATATCCTGCTGATCAAA<br>TATGAAGATCTGATGGTTGATCCGGTTGTGCAGCTGAAAGCACTGGCAGAATTTCTGAAA<br>CTGCCGTTTACCAAAGAAGAAGAGAAAGACAACATTATCCACGGCATTATTAACGCCTGT<br>AGCTTTCAGACCGTTAAAGACAGCAAAATGTATGAAAGCGGTAATACCGAACTGCTGCAG<br>GTTCAGGTGAATAACACCACCTTTCTGCGCAAAGGTAAAACCAATGATTGGGAAAATTAT<br>CTGACCCCGAAAATGGCAGAACGTCTGGACCTGCTGACCGTGCAGAAATTTGCAGATACC<br>GATCTGATCTAACAAGCTTGCGGCCGCATAATGCTTA
**CBJBB21**	*Zostera marina* KMZ74024.1	TAGAAATAATTTTGTTTAACTTTAAGAAGGAGATATACCATGGACGAAAGCAATACCCTG<br>AGCAGCATTAGCACCGATGAACAGCATAAAAGCATCGAAGAAAGCTTCGCCAAATACAAA<br>CAGCTGATTGCAAGCACCTATCCGCGTTGTGATCTGCCGCTGCAGCCGACACTGTTTCAG<br>CATAAAGATCATATGTGGTTCAGCACCTATGAACTGATGGTTAGCACCCTGGTTGCACAG<br>GATCATCTGGTTAGCCGTCCGACCGATGTTTTTGTTACCAGCTTTCCGAAAAGCGGCACC<br>ACCTGGCTGAAAGGTCTGGTTAGTAGCATTAGTACCCGTAACGATATGGATAAAGTTAAT<br>AGCCTGGCACCGCATCATCAGATTCCGTTTCTGGAATTTCATGTGTATCCGTCCGATGAT<br>CAGATCATGGATATTGATAGCCTGCAGAGTCCGCGTTTTCTGAGCACCCATATTCCGTAT<br>CCGAGCCTGCCGAAAAGCATGATTGATAGCGGTTGTCGTATTGTGTATATTTGGCGTGAT<br>CCGAAAGCAACCTTTGTGAGCTTTTTTCATTTTAGCAATCAGATCCGCACCAAATATGGT<br>ACAGCACTGAGCAGTATTGAAGATCGTCTGAAATGGTTTTGTGATGGCTATTGTTGGTTT<br>GGTCCGTATTGGGATCATGTGTTAGGTTATTGGAACGCCAAAAAAAACGGTGGCAATATC<br>CTGTTCCTGAAATATGAGGATATGATGGAAGATCCGTGCAGCCATATCAAAACCCTGGCA<br>GAATTTATGGGTGTTCCGTTTACCGAACAAGAAGAAAAAGAAGGCGTTATGCAGGCAATT<br>ATTGCAAGCTGTAGCTTCGACAAAATGAAAGATTCCGACGTGAACAAAATCGGCAAAACC<br>ATGCTGCATGTTGAAGTGGATAACAATTCCTTTTTTCGCGAAGGCAAAACGAATGACTGG<br>GTGAATTATCTGACACCGGAAATGGTTGAAAAACTGGATCGTATTACCAAAGAACGTTTT<br>GCAGATACCGATCTGATTCCGTAACAAGCTTGCGGCCGCATAATGCTTA
**CBJBB22**	*Zostera marina* KMZ64284.1	TAGAAATAATTTTGTTTAACTTTAAGAAGGAGATATACCATGGATCCGCTGATGGATGAA<br>TGTGAAGTTTTTAGTGCCGGTGTTGATGATGATCAGCAAGAAACCATTAAAGAGAGCTAC<br>GCAAAATATCGTCAGCTGGTTGCCACCTTTCCGCGTTGTACCTGTCTGAGCAGCGAATTT<br>GCACCGCTGGAAGCAACCTTTTTTCAGCATAAAGAACATGGCTGGATCAACATGTTTCCG<br>ATTATGGTTAATAGCCTGGTTGCCCAGAAACATTTTGTTGGTCGTCCGACCGATGTTTTT<br>GTTAGCAGCCTGCCGAAAAGCGGCACCGTTTGGCTGAAAGATCTGGTTTGTAAAATTACC<br>AGCCGTGGTGGTGATCTGGATAACAAAAATGACCTGTTTAGTCCGCATCACAAAGTTCCG<br>TTTCTGGAACTGCAGGTTTATGCAAGCGAAGATAATATGCTGGATATTGATAGCCTGCCG<br>TCACCGCGTCTGCTGAGCACCCATATTCCGTATCCGAGCCTGCCTGCAAGCCTGATTAAT<br>AGCGGTTGTCCGATTGTGTATATTTGGCGTGATCCGAAAGCAATCTTTGTTAGCGATTGG<br>CACTTTTTCAATAACATCGTTCCGAGCAAACTGGGCGAAAATATTCCGACCATTGAAGAA<br>CGTTTTGAGTGCTTTTGTAACGGCTATAGCAAATTTGGTCCGTATTGGGATCATGTGTTA<br>GGTTATTGGAACGCCAAAAAAAACGGTGCGAATATCCTGCTGATCAAATATGAAGATCTG<br>ACCGCAGATCCGGTTGTGCAGCTGAAAGCACTGGCAGAATTTCTGAAACTGCCGTTTACC<br>GAAGAGGAAGAGAAAGACAACGTTATCAAAGATATTGTTACCGCCTGTAGCTTCCAGATG<br>GTTAAAGATAGCAAAATGTATGAAAGCGGTGTGACCAAAGTTCTGCATCGTCAGATTAAA<br>AACACCATGTTTCTGCGTGAAGGCAAAACCAATGATTGGGAAAATTATCTGACCCCGAAA<br>ATGGCAGAACGTCTGGATCTGATTACCATTCAGCGTTTTGCAGATACCGATCTGATCTAA<br>CAAGCTTGCGGCCGCATAATGCTTA
**CBJBB23**	*Zostera marina* KMZ72298.1	TAGAAATAATTTTGTTTAACTTTAAGAAGGAGATATACCATGAGCAAGGCAATGATTAGC<br>GCAAGCCAGAGCCATAGCTTTATCTATAAAGCAAACACCAAACTGAAGCTGAAAAGTCCG<br>CAGAGCCCGATGGCAGAATGTGAAGTTGTTAGCGGTCTGGTTCTGACCGATGAAATTGCA<br>GATGATGATCGTCAAGAAACCATCAAAGAAAGCTTTGCCAAATATCGTCAGCTGGTTGCC<br>ACCTTTCCGCGTTGTACCTATCTGAGCAGCGAATGTGTTCCGCTGGAAGATGCATTTTTT<br>CAGCATAAAGAACAAGGTTGGATCAGCCGTTTTCATGTTATGGTTAATTGTCTGGTTGCC<br>CAGAAACATTTTGTTGGTCGTCCGACCGATGTTTTTCTGGGTAGCCTGCCGAAAAGCGGC<br>ACCGTTTGGCTGAAAGATCTGGTGTATAAAATCACCCGTCGTGGTGATGATCAGGATCAT<br>AAAAATGATCTGCTGAGTCCGCATCAGAAAGTTCCGTTTCTGGAACTGCAGGTTTATGCA<br>AGCGAAGCAAATGTTCTGGATATTGATAGTCTGCCGTCACCGCGTCTGCTGAGCACCCAT<br>ATGCCGTATCCGAGCCTGCCTGCAAGCCTGATTGATAGCGGTTGTCCGATTGCATATATT<br>TGGCGTGATCCGAAAAGCATCTTTGTTAGCGATTGGCACTACTTCAACAAAATCATTCCG<br>AGCAAACCGGGTACAAATCTGCCGAGCCTGACCATTAATGAAAAATTTGAATGCTTCTGC

		AACGGCTACAGCATTTTTGGTCCGTATTGGGATCATGTGTTAGGTTATTGGAACGCCAAA<br>AAAAACGGTGCGAATATCCTGCTGATCAAATATGAAGATCTGATGGATGATCCGATCGTT<br>CATCTGAAAGCACTGGCAGAATTTCTGAAAGTGCCGTTTACCGAAGAAGAAGAGAAAGAT<br>AACATTATCCAGGGCATTATTACCGCCTGTAGCTTTAGCAAAGTGAAAGACAGCAAAATG<br>TATAACAGCGGCAACACCAAAATTCTGGGCTATCAGATTCATAACACCATGTTTCTGCGT<br>GAAGGCAAAACCAATGATTGGGAAAATTATCTGACACCGGAAATGGCAGATCGTCTGGAC<br>CTGCTGACCGTGAAAAAATTCGCAGATACCGATCTGATCTAACAAGCTTGCGGCCGCATA<br>ATGCTTA
**CBJBB24**	*Zostera marina*<br>KMZ72292.1	TAGAAATAATTTTGTTTAACTTTAAGAAGGAGATATACCATGGCCGAATGTGAAGTTGTT<br>AGCGGTCTGGTTTTTACCGATGAAATCAGTGATGATGATCGCCAGAAAACCATCAAAGAA<br>AGCTTTGCAAAATATCGTCGTCTGGTTGCCACCTTTCCGCGTTGTACCTGTCTGAGCAGC<br>GAATGTGTTCCGCTGGAATATGCATTTTTTCAGCATAAAGAACAAGGCTGGATTAGCCGT<br>TTTCCGACCATGGTTAATTGCCTGGTTGCACAGAAACATTTTGTTGGTCGTCCGACCGAT<br>GTTTTTCTGGGTAGCCTGCCGAAAAGCGGCACCGTTTGGCTGAAAGATCTGGTGTATAAA<br>ATCACCCGTCGTTGTGGTGATCAGGATTGTAAAAATGATCTGCTGAGTCCGCATCAGAAA<br>GTTCCGTTTCTGGAAAATCAGGTTTATGCCAGCGAAGATAATGTGCTGGATATTGATAGT<br>CTGCCGTCACCGCGTCTGCTGAGCACCCATATGCCGTATCCGAGCCTGCCTGCAAGCCTG<br>ATTGATAGCGGTTGTCCGATTGTGTATATTTGGCGTGATCCGAAAAGCATCTTTGTTAGC<br>GATTGGCACTTCTTCAACAAAATTCTGCCGAGCAAACCGGGTACAAATCTGCCGAGTCTG<br>ACCATTAATGAAAAATTTGAATGCTTTTGCAACGGCTATAGCTATTTTGGTCCGTATTGG<br>GATCATGTGTTAGGTTATTGGAACGCCAAAAAAAACGGTGCGAACATCCTGTTCATCAAA<br>TATGAAGATCTGATGGATGATCCGATCGTTCATCTGAAAGCACTGGCCGAATTTCTGAAA<br>ATTCCGTTTAGCGAAGAGGAAGAGAAAGACAACATTATTCAGGGTATTATTACCGCCTGC<br>AGCTTCAGCAAAGTTAAAGATAGCAAAATGTACAACAGCGGCAACACCAAATTTCTGGAT<br>TATCAGGTGCATAACACCATGTTTCTGCGTGAAGGTAAAACCAATGATTGGGCAAATTAT<br>CTGACCCCGAAAATGGCAGATCGTCTGGACCTGCTGACCGTGAAAAAATTCGCAGATACC<br>GATCTGATCTAACAAGCTTGCGGCCGCATAATGCTTA
**CBJBB25**	*Zostera marina*<br>KMZ72296.1	TAGAAATAATTTTGTTTAACTTTAAGAAGGAGATATACCATGGCCGAATGTGAAGTTGTT<br>AGCCGTCTGGTTTTTACCGATGAAGTTGCAGATGATGATCGCCAAGAAACCATCAAAGAA<br>AGCTTTGCAAAATATCGTCAGCTGGTTGCCACCTTTCCGCGTTGTACCTGTCTGAGCAGC<br>GAATGTGTTCCGCTGGAAACCGCATTTTTTCAGCATAAAGAACAAGGTTGGATTAGCCGT<br>TTTCCGGCAATGGTTAATTGTCTGGTTGCACAGAAACATTTTGTTGGTCGTCCGACCGAT<br>GTTTTTCTGGGTAGCCTGCCGAAAAGCGGCACCGCATGGCTGAAAGATCTGGTGTATAAA<br>ATCACCCGTCGTGGTGGTGATCAGGATCATAAAAATGATCTGCTGAGTCCGCATCAGAAA<br>GTTCTGTTTCTGGAACTGCAGGTTTATGCCAGCGAAGATAATGTTCTGGATATCGATCTG<br>CTGCCGAGTCCGCGTCTGCTGAGCACCCATATGCCGTATCCGAGCCTGCCTGCAAGCCTG<br>ATTGATAGCCGTTGTCCGATTGTGTATATTTGGCGTGATCCGAAAAGCATCTTTGTTAGC<br>GATTGGCACTTCTTCAACAAAATCATTCCGAGCAAACCGGATGCAAATCTGCCGAGCCTG<br>ACCATTAATGAAAAATTTGAATGCTTCTGCGACGGCTATAGCATTTTTGGTCCGTATTGG<br>GATCATGTGTTAGGTTATTGGAACGCCAAAAAAAACGGTGCCAATATCCTGCTGATCAAA<br>TATGAGGATATGATGGATGATCCGATCGTTCATGTTAAAGCACTGGCAGAATTTCTGAAA<br>ATCCCGTTTACCGAAGAGGAAGAGAAAGACAACGTTATTCAGGGTATTATCATTGCCTGC<br>AGCTTCAGCAAAGTGAAAGATAGCAAAATGTATAACAGCGGCAACACCAAATTTCTGGAT<br>TATCAGGTTCACAACACCATGTTTCTGCGTGAAGGTAAAACCAATGATTGGGCAAATTAT<br>CTGACCCCGAAAATGGCAGATCGTCTGGACCTGCTGACCGTGAAAAAATTCGCAGATACC<br>GATCTGATCTAACAAGCTTGCGGCCGCATAATGCTTA
**CBJBB26**	*Zostera marina*<br>KMZ76265.1	TAGAAATAATTTTGTTTAACTTTAAGAAGGAGATATACCATGGCAGGTATTCTGCCGGTT<br>GAACGTTGTTTTGCACCGAAAAAACTGGAACAGACCGAAGAGGATAGCAAAATGTATAAA<br>CGCTATGCCGAAATCGTTTATAGCCTGCCGCTGGTTGATAGCTGGGGCACCAAACTGGCC<br>ATGTATAAAGGTTTTTGGCTGCTGCATGAAATTCTGCCTGGTATTATGGCATTTCACGAT<br>CATTTTAAAGGTCGTCGCACCGATATTATTCTGACCGCAATTCCGAAAGCAGGCACCACC<br>TGGACCAAAGCACTGGCATTTGCAATTCTGACCCGTGAAGTTAATCATCCGAGCAGCCCG<br>ACACATCCGCTGCTGGGTTTTAATCCGCATAGCTGTGTTACCACACTGGAATATCTGTAT<br>ATGGGTCGTGAAAATCTGATGCCGGATGCAGATGTTCTGAAAGAAAGTCCGCGTCTGTTT<br>GCAACCCATATGCCGTTTAGCCTGCTGCCGAAAAGCATTGTTGAAAGCCGTGCAAAAATC<br>ATTAATGTGAGCCGTGAACGTAAAAGCACCTTTGTTAGCAACTGGAAATTCTTTAACGAC<br>ATTTGGAGCGAAAACGTGCCTAAAAATCTGGATCTGGAAAAATGCGTTGAACTGTTTGCC<br>AGCGGTATTAGCTATTGTGGTCCGGAATGGGTTCATCGTGCAGAATATACCAATGCACTG<br>AGCACCAATAGCAATCTGCTGCTGCTGAGCTATGAAGAGATGATGGAAAAACCGGTGGAA

		AACGTGAAAAAAATGGCCGATTTTATGGGTTGTCGCTTTACCGATGATGAAGTGAAACTG<br>GGTATTGTGGATGAAATTGTTAAACTGTGCAGCTTCGACAACCTGAAAAAACAGCAGGTT<br>AACAAAATTGGTCGCAGCTACAGCAAAATGAACAACAAACACTTTTTTCGCAAAGGCGAA<br>GTTAATGATTGGGCGAATCATCTGACACCGGAAATGGTTATTAATCTGGATAAAGCAGGT<br>CAGGGCACCAACTAACAAGCTTGCGGCCGCATAATGCTTA
**CBJBB27**	*Zostera marina*<br>KMZ73756.1	TAGAAATAATTTTGTTTAACTTTAAGAAGGAGATATACCATGGATCCGAGCATGAATGAA<br>TATGAAGTTTTTAGCGGTGTGGTGGATGATGATCAGCAAGAAACCATTAAAGAAAGCTAT<br>GCCAAATATCGTCAGCTGGTTGCAGCATTTCCGCGTTGTACCAGCCTGAGCAGCGAATTT<br>GCACCGCTGGAAGCAGCATTTTTTCAGCACAAAGAATACAAATGGATCAGCCAGTTTAGC<br>ACCATGGTTAATTGTCTGGTTGCCGAAAAACATTTTGTTGGTCGTCCGATTGATGTTTTT<br>GTGGGTAGCTTTCCGAAAAGCGGCACCGTTTGGCTGAAAGATCTGGTGTATAAAATCACC<br>CGTCATGGTGGTAATCAGGATCATAAAAATGATCTGCTGAGTCCGCATCAGAAAGTTCCG<br>TTTCTGGAACTGCAGATTTATAGCAGCGAAGATCATGTGCTGGATATTGATAGCCTGCCG<br>AGTCCGCGTGTTCTGAGCACCCATATTCCGTATCCGAGTCTGCCGACCAGTCTGATTGAT<br>AGCGGTTGTCCGATTGTGTATATTTGGCGTGATCCGAAAGCAGTTTTTGTTAGCGATTGG<br>CACTTCTTCAACAAAATCATTCCGAGCGAACCGGGTACAAATCTGCCGAGCTTTACCATT<br>AACAAAAAGTTTGAATGCTTTTGCAACGGCTACTCCATTTTTGGTCCGTATTGGGATCAT<br>GTGTTAGGTTATTGGAACGCCAAAAAAAACGGCGTGAATATCCTGCTGATCAAATATGAA<br>GATCTGATGGCAGATCCGGTTGTGCAGCTGAAAGCACTGGCAGAATTTTAACAAGCTTGC<br>GGCCGCATAATGCTTA
**CBJBB28**	*Zostera marina*<br>KMZ69186.1	TAGAAATAATTTTGTTTAACTTTAAGAAGGAGATATACCATGGACTTTAGCGTTAGCCAT<br>CACCGTAAACTGTTTAGCGGCACCAAAGTTTTTCGTGGTAGCAGCCATCGTTGTGTTTAT<br>CGTCTGACACCGCAGATTCGTGATGGTCTGGTTGAAGCACTGCAGAAAAAAGTTGGTAGC<br>AGTGATGAAGCAAAACTGACCAAATTTTCCCTGAAAATCGTGTTCGTTGTGAAAGATCTG<br>GTGTACAAAATCAAAAGCCGTGGTGGTAATCAGGACCACAAAAAAGATCTGCTGAGTCCG<br>CATCAGAAAGTTCCGTTTCTGGAACTGCAGGTTTATGCAAGCGAAGATCATGTTCTGGAT<br>ATTGATAGCCTGCCGAGTCCGCGTCTGCTGAGCACCCATATTCCGTATCCGAGTCTGCCG<br>ACCAGCCTGATTGATAGCGGTTGTAGCATTGTGTATATTTGGCGTGATCCGAAAGCCTTT<br>GAATGCTTTTGTAATGGCTATAGCATCTTCGGTCCGTATTGGGATCATGTTTTAGGTTAT<br>TGGAACGCCAAAAAAAACGGTGCCAATATCCTGCTGATCAAATATGAAGATCTGATGGTT<br>GATCCGGTTGCGCAGCTGAAAATTCTGGCGGAATTTCTGAAACTGCCGTTTACCGAAGAA<br>GAGGAAGAGGATAACATTATCCATGGCATTATTACCGCATGCAGCTTTCAGATGGTTAAA<br>GACAGCAAAATGTATGAAAGCGGCAATACCGAACTGCTGCAGGTTCAGGTGAATAACACC<br>ACCTTTCTGCGTGAACGTAAAACCAATGATTGGGAGAATTACCTGATTCTGAAAATGGCA<br>GAACGCCTGAATCTGATTACCGTTCAGCGTTTTGCAGATACCGATCTGATTTAACAAGCT<br>TGCGGCCGCATAATGCTTA
