# Supplementary material for: Production of zosteric acid and other sulfated phenolic biochemicals in microbial cell factories
Source: Nat Commun. 2019 Sep 6;10:4071. doi: 10.1038/s41467-019-12022-x (PMC6731281; doi:10.1038/s41467-019-12022-x)
Supplement: Supplementary file 6 — Supplementary Data 2 [file 41467_2019_12022_MOESM6_ESM.pdf]

Supplementary Data 2

Production of zosteric acid and other sulfated phenolic biochemicals in microbial cell factories

Jendresen *et al*

Supplementary Data 2: Oligonucleotides

**Oligo-nucleotide**	**Gene**	**Direction**	**Sequence**	**Restriction site[a]**	**Use[b] and references**
PPGK1_fw	PPGK1 promoter	Forward	CGTGCGAUGGAAGTACCTTCAAAGA		Sc[1]
PPGK1_rv	PPGK1 promoter	Reverse	ATGACAGAUTTGTTTTATATTTGTTG		Sc[1]
PTEF1_fw	PTEF1 promoter	Forward	ACCTGCACUTTGTAATTAAAACTTAG		Sc[1]
PTEF1_rv	PTEF1 promoter	Reverse	CACGCGAUGCACACACCATAGCTTC		Sc[1]
CBJP470	*H. sapiens* SULT1A1	Forward	TAGAAATAATTTTGTTTAACTTTAAGAAGGAGATATACCATGGAACTGATTCAGGATACCAG		Ec
CBJP471	*H. sapiens* SULT1A1	Reverse	TAAGCATTATGCGGCCGCAAGCTTGTTACAGTTCGCTACGAAAGCTC		Ec
CBJP472	*R. norvegicus* SULT1A1	Forward	TAGAAATAATTTTGTTTAACTTTAAGAAGGAGATATACCATGGAGTTCTCCCGTCCAC		Ec
CBJP473	*R. norvegicus* SULT1A1	Reverse	TAAGCATTATGCGGCCGCAAGCTTGTCATAGTTCACAACGAAACTTGAA		Ec
CBJP474	*D. melanogaster* dmST1	Forward	TAGAAATAATTTTGTTTAACTTTAAGAAGGAGATATACCATGCCCCAGTCGAGCTTCTT		Ec
CBJP475	*D. melanogaster* dmST1	Reverse	TAAGCATTATGCGGCCGCAAGCTTGTTACGTGGACGCAAACTTGCT		Ec
CBJP478	*D. melanogaster* dmST3 - variant A	Forward	TAGAAATAATTTTGTTTAACTTTAAGAAGGAGATATACCATGAATCGGGTTCAGGTCAC		Ec
CBJP479	*D. melanogaster* dmST3 - variant A	Reverse	TAAGCATTATGCGGCCGCAAGCTTGTCAAACAGCTTGACCTTGCTTTA		Ec
CBJP481	*D. melanogaster* dmST4	Forward	TAGAAATAATTTTGTTTAACTTTAAGAAGGAGATATACCATGGAAAATACGCCTCTCAA		Ec
CBJP482	*D. melanogaster* dmST4	Reverse	TAAGCATTATGCGGCCGCAAGCTTGTCATATGGTCTTGAATGATT		Ec
CBJP491	*E. coli cysD*	Forward	CATCTTAGTATATTAGTTAAGTATAAGAAGGAGATATACATATGGATCAAATACGACTTACTCAC		Ec
CBJP492	*E. coli cysC*	Reverse	TGGCCGGCCGATATCCAATTGATCAGGATCTGATAATATCGTTCTG		Ec
CBJP495	E. coli *cysQ*	Forward	CATCTTAGTATATTAGTTAAGTATAAGAAGGAGATATACATATGTTAGATCAAGTATGCCAGC		Ec
CBJP496	E. coli *cysQ*	Reverse	TGGCCGGCCGATATCCAATTGATTAGTAAATAGACACTCTGAACCC		Ec
CBJP497	*E. coli cysDNC*	Reverse	TCAGGATCTGATAATATCGTTCTG		Ec
CBJP498	*E. coli cysC*-P_{cysQ}	Forward	CAGAACGATATTATCAGATCCTGATAAGTTAACACCGCTCACAGAGACGAGGTGGAGAA		Ec
CBJP499	*E. caballus* SULT1A1	Forward	TAGAAATAATTTTGTTTAACTTTAAGAAGGAGATATACCATGGAGCTGATCCAGGACACC		Ec
CBJP500	*E. caballus* SULT1A1	Reverse	TAAGCATTATGCGGCCGCAAGCTTGTCACACCTCTGAGCGGAAGC		Ec

CBJP501	*G. gallus* domesticus SULT1E1	Forward	TAGAAATAATTTTGTTTAACTTTAAGAAGGAGATATACCATGGGGAATGATGAGGTGATCAG		Ec
CBJP502	*G. gallus domesticus* SULT1E1	Reverse	TAAGCATTATGCGGCCGCAAGCTTGTTACTCTGTCTATTGCAATTTATTACAGG		Ec
CBJP503	*C. lupus familiaris* SULT1A1	Forward	TAGAAATAATTTTGTTTAACTTTAAGAAGGAGATATACCATGGAGGACATTCCCGAC		Ec
CBJP504	*C. lupus familiaris* SULT1A1	Reverse	TAAGCATTATGCGGCCGCAAGCTTGTCACAGCTGTGTGCGGAAGC		Ec
CBJP505	*S. scrofa domesticus* SULT1A1	Forward	TAGAAATAATTTTGTTTAACTTTAAGAAGGAGATATACCATGGAGCCGGTCCAGGAC		Ec
CBJP506	*S. scrofa domesticus* SULT1A1	Reverse	TAAGCATTATGCGGCCGCAAGCTTGTCACAGCTCAGAGCGGAAGC		Ec
CBJP513	*G. gallus domesticus* SULT1B1 - clone1	Forward	TAGAAATAATTTTGTTTAACTTTAAGAAGGAGATATACCATGGGCACAGTGGATGCCT		Ec
CBJP514	*G. gallus domesticus* SULT1B1 - clone1	Reverse	TAAGCATTATGCGGCCGCAAGCTTGTCAGATGTGTGTGCGGAAGC		Ec
CBJP515	*G. gallus domesticus* SULT1B1-predicted - clone1	Forward	TAGAAATAATTTTGTTTAACTTTAAGAAGGAGATATACCATGGCCACGGTGGACAG		Ec
CBJP516	*G. gallus domesticus* SULT1B1-predicted - clone1	Reverse	TAAGCATTATGCGGCCGCAAGCTTGTCAGATTTCTGTGCGGAAGC		Ec
CBJP517	*G. gallus domesticus* SULT1C1	Forward	TAGAAATAATTTTGTTTAACTTTAAGAAGGAGATATACCATGGCCCTGGATAAAATGG		Ec
CBJP518	*G. gallus domesticus* SULT1C1	Reverse	TAAGCATTATGCGGCCGCAAGCTTGTCACAATTCCATGCGAAAAACTAG		Ec
CBJP519	*G. gallus domesticus* SULT2B1-predicted	Forward	TAGAAATAATTTTGTTTAACTTTAAGAAGGAGATATACCATGCCGGTGCACTACGTGAC		Ec
CBJP520	*G. gallus domesticus* SULT2B1-predicted	Reverse	TAAGCATTATGCGGCCGCAAGCTTGTCAGTTAGGTCAGGATCCTGATCTC		Ec
CBJP533	*R. norvegicus* SULT1A1 (codon-opt)	Forward	TAGAAATAATTTTGTTTAACTTTAAGAAGGAGATATACCATGGAATTTTCACGTCC		Ec
CBJP534	*R. norvegicus* SULT1A1 (codon-opt)	Reverse	TAAGCATTATGCGGCCGCAAGCTTGTTACAGTTCACAACGAAATTTG		Ec
CBJP633	SULT1A1$_{Rno}$	Forward	AGTGCAGGUAAAACAATGGAGTTCTCCCGTCCA		Sc
CBJP634	SULT1A1$_{Rno}$	Reverse	CGTGCGAUTCATAGTTCACAACGAAACTTG		Sc
CBJP637	TAL$_{Ses}$	Forward	ATCTGTCAUAAAACAATGACCCAGGTTGTTGAACG		Sc
CBJP638	TAL$_{Ses}$	Reverse	CACGCGAUTCAGCCAAAATCTTTACCATCTGC		Sc
CBJP647	TAL$_{Fjo}$	Forward	ATCTGTCAUAAAACAATGAACACCATCAACGAATATCTG		Sc
CBJP648	TAL$_{Fjo}$	Reverse	CACGCGAUTCAATTGTTAATCAGGTGGTCTTTTACTTTCTG		Sc

CBJP651	TAL$_{Fjo, Sc}$	Forward	ATCTGTCAUAAAACAATGAACACCATCAACGAATACTTG		Sc
CBJP652	TAL$_{Fjo, Sc}$	Reverse	CACGCGAUTCAGTTGTTAATCAAGTGATCCTTAACTTTTTGG		Sc
CBJP731	SULT1A1$_{Rno, Sc}$	Forward	AGTGCAGGUAAAACAATGGAATTTTCACGTCCGC		Sc
CBJP732	SULT1A1$_{Rno, Sc}$	Reverse	CGTGCGAUTCACAGTTCACAACGAAATTTGAA		Sc
CBJP891	*cysZ*	Forward	TTAAAAGCTTGGGATTGGTCAAAAGGAGCTCATCC	*Hin*dIII	Ec
CBJP892	*cysZ*	Reverse	AATAGCGGCCGCTTACCGCCACATCGCGTGTTTAT		Ec
CBJP893	*cysP*	Forward	TTAAAAGCTTAGAAAGTCATTAAATTTATAAGGGTGCGCA	*Hin*dIII	Ec
CBJP894	*cysA*	Reverse	AATAGCGGCCGCTCAGGCGCTTTGTGCGAGAGC	*Not*I	Ec
CBJP908	*cysU*	Forward	TGATGTTTGCTGTCTCCTCCA		Ec
CBJP910	*sbp*	Forward	AGCTCGGCGCGCCTGCAGGTCGACAAGCTTCATCACAACACAACATAAGAGAGTC	*Hin*dIII	Ec
CBJP911	*sbp*	Reverse	GCACGCGTCTGGAGGAGACAGCAAACATCAGCGTTTGCTGATCTGATCG		Ec
CBJP912	*cysP$_{Bsu}$*	Forward	TTAAAAGCTTATGCGGACTGCATGAATAAGGAGC	*Hin*dIII	Ec
CBJP913	*cysP$_{Bsu}$*	Reverse	AATAGCGGCCGCTCAGATTCCCCCGCCTTGTTCG	*Not*I	Ec

[a] Underlining indicates restriction sites.
[b] Ec, expression in *E. coli*; Sc, expression in *S. cerevisiae*.

1. Jensen, N. B. *et al.* EasyClone: Method for iterative chromosomal integration of multiple genes in Saccharomyces cerevisiae. *FEMS Yeast Res.* **14**, 238–248 (2014).
