# Supplementary material for: Production of zosteric acid and other sulfated phenolic biochemicals in microbial cell factories
Source: Nat Commun. 2019 Sep 6;10:4071. doi: 10.1038/s41467-019-12022-x (PMC6731281; doi:10.1038/s41467-019-12022-x)
Supplement: Supplementary file 7 — Supplementary Data 3 [file 41467_2019_12022_MOESM7_ESM.pdf]

Supplementary Data 3

Production of zosteric acid and other sulfated phenolic biochemicals in microbial cell factories

Jendresen *et al*

Supplementary Data 3: Plasmids

Name	Parent vector	Description	Markers[a]	Reference
pETDuet-1			Amp^r	Novagen
pCDFDuet-1			Sp^r	Novagen
pRSFDuet-1			Kan^r	Novagen
pCfB132	pESC-URA	Episomal replication vector with USER cassette	Amp^r, URA	[1]
pCBJ215	pCDFDuet-1	pCDFDuet-1 MCS2::TAL_{Rsp}	Sp^r	[2]
pCBJ228	pCDFDuet-1	pCDFDuet-1 MCS2::TAL_{Fjo}	Sp^r	[2]
pCBJ255	pETDuet-1	pETDuet-1 MCS1::*H. sapiens* SULT1A1	Amp^r	This work
pCBJ256	pETDuet-1	pETDuet-1 MCS1::*R. norvegicus* SULT1A1 ($SULT1A1_{Rno}$)	Amp^r	This work
pCBJ258	pETDuet-1	pETDuet-1 MCS1::*D. melanogaster* dmST1	Amp^r	This work
pCBJ259	pETDuet-1	pETDuet-1 MCS1::*D. melanogaster* dmST1 - clone2	Amp^r	This work
pCBJ260	pETDuet-1	pETDuet-1 MCS1::*D. melanogaster* dmST3 - variant A	Amp^r	This work
pCBJ261	pETDuet-1	pETDuet-1 MCS1::*D. melanogaster* dmST4	Amp^r	This work
pCBJ262	pETDuet-1	pETDuet-1 MCS1::*E. caballus* SULT1A1	Amp^r	This work
pCBJ263	pETDuet-1	pETDuet-1 MCS1::*G. gallus domesticus* SULT1E1	Amp^r	This work
pCBJ264	pETDuet-1	pETDuet-1 MCS1::*C. lupus familiaris* SULT1A1	Amp^r	This work
pCBJ265	pETDuet-1	pETDuet-1 MCS1::*S. scrofa domesticus* SULT1A1	Amp^r	This work
pCBJ269	pETDuet-1	pETDuet-1 MCS2::*cysQ*	Amp^r	This work
pCBJ271	pRSFDuet-1	pRSFDuet-1 MCS2::*cysDNC*	Kan^r	This work
pCBJ272	pRSFDuet-1	pRSFDuet-1 MCS2::*cysDNCQ*	Kan^r	This work
pCBJ282	pCfB132	pCfB132::PPGK1->$TAL_{Fjo, Sc}$	Amp^r, URA	[2]
pCBJ283	pCfB132	pCfB132::$SULT1A1_{Rno}$<-PTEF1	Amp^r, URA	This work
pCBJ284	pCfB132	pCfB132::$SULT1A1_{Rno, Ec}$<-PTEF1	Amp^r, URA	This work
pCBJ285	pCfB132	pCfB132::$SULT1A1_{Rno, Ec}$<-PTEF1-PPGK1->TAL_{Ses}	Amp^r, URA	This work
pCBJ287	pCfB132	pCfB132::$SULT1A1_{Rno, Ec}$<-PTEF1-PPGK1->TAL_{Fjo}	Amp^r, URA	This work
pCBJ289	pCfB132	pCfB132::$SULT1A1_{Rno, Ec}$<-PTEF1-PPGK1->$TAL_{Fjo, Sc}$	Amp^r, URA	This work
pCBJ297	pCDFDuet-1	pCDFDuet-1 MCS2::TAL_{Rca}	Sp^r	[2]
pCBJ320	pETDuet-1	pETDuet-1 MCS1::*G. gallus domesticus* SULT1B1 - clone1	Amp^r	This work
pCBJ321	pETDuet-1	pETDuet-1 MCS1::*G. gallus domesticus* SULT1B1 - clone2	Amp^r	This work
pCBJ322	pETDuet-1	pETDuet-1 MCS1::*G. gallus domesticus* SULT1B1 - clone3	Amp^r	This work
pCBJ323	pETDuet-1	pETDuet-1 MCS1::*G. gallus domesticus* SULT1B1-predicted - clone1	Amp^r	This work
pCBJ324	pETDuet-1	pETDuet-1 MCS1::*G. gallus domesticus* SULT1B1-predicted - clone2	Amp^r	This work

pCBJ325	pETDuet-1	pETDuet-1 MCS1::*G. gallus domesticus* SULT1C1	Amp^r	This work
pCBJ327	pETDuet-1	pETDuet-1 MCS1::*G. gallus domesticus* SULT2B1-predicted	Amp^r	This work
pCBJ329	pETDuet-1	pETDuet-1 MCS1::*R. norvegicus* SULT1A1 (codon-opt)	Amp^r	This work
pCBJ332	pETDuet-1	pETDuet-1 MCS1::SULT1A1$_{Rno}$-*cysZ*	Amp^r	This work
pCBJ333	pRSFDuet-1	pRSFDuet-1 MCS1::*cysZ* MCS2::*cysDNCQ*	Kan^r	This work
pCBJ334	pRSFDuet-1	pRSFDuet-1 MCS1::*cysPUWA* MCS2::*cysDNCQ*	Kan^r	This work
pCBJ336	pETDuet-1	pETDuet-1 MCS1::*C. elegans* SSU-1	Amp^r	This work
pCBJ337	pETDuet-1	pETDuet-1 MCS1::*D. rerio* SULT1ST1	Amp^r	This work
pCBJ338	pETDuet-1	pETDuet-1 MCS1::*D. rerio* SULT4A1	Amp^r	This work
pCBJ339	pETDuet-1	pETDuet-1 MCS1::*D. rerio* SULT6B1	Amp^r	This work
pCBJ340	pETDuet-1	pETDuet-1 MCS1::*A. thaliana* AtSOT12	Amp^r	This work
pCBJ341	pETDuet-1	pETDuet-1 MCS1::*Streptomyces* Cpz8	Amp^r	This work
pCBJ342	pETDuet-1	pETDuet-1 MCS1::*Streptomyces* LipE	Amp^r	This work
pCBJ343	pETDuet-1	pETDuet-1 MCS1::*S. punctatus* DAOM BR117 SPPG_07427	Amp^r	This work
pCBJ344	pETDuet-1	pETDuet-1 MCS1::*H. ochraceum* DSM 14365 Hoch_6098	Amp^r	This work
pCBJ345	pETDuet-1	pETDuet-1 MCS1::*R. radiotolerans* RradSPS_0172	Amp^r	This work
pCBJ346	pETDuet-1	pETDuet-1 MCS1::*Zostera marina* KMZ76263.1	Amp^r	This work
pCBJ347	pETDuet-1	pETDuet-1 MCS1::*Zostera marina* KMZ64288.1	Amp^r	This work
pCBJ348	pETDuet-1	pETDuet-1 MCS1::*Zostera marina* KMZ76264.1	Amp^r	This work
pCBJ349	pETDuet-1	pETDuet-1 MCS1::*Zostera marina* KMZ59959.1	Amp^r	This work
pCBJ350	pETDuet-1	pETDuet-1 MCS1::*Zostera marina* KMZ74024.1	Amp^r	This work
pCBJ351	pETDuet-1	pETDuet-1 MCS1::*Zostera marina* KMZ64284.1	Amp^r	This work
pCBJ352	pETDuet-1	pETDuet-1 MCS1::*Zostera marina* KMZ72298.1	Amp^r	This work
pCBJ353	pETDuet-1	pETDuet-1 MCS1::*Zostera marina* KMZ72292.1	Amp^r	This work
pCBJ354	pETDuet-1	pETDuet-1 MCS1::*Zostera marina* KMZ72296.1	Amp^r	This work
pCBJ355	pETDuet-1	pETDuet-1 MCS1::*Zostera marina* KMZ76265.1	Amp^r	This work
pCBJ356	pETDuet-1	pETDuet-1 MCS1::*Zostera marina* KMZ73756.1	Amp^r	This work
pCBJ357	pETDuet-1	pETDuet-1 MCS1::*Zostera marina* KMZ69186.1	Amp^r	This work
pCBJ361	pETDuet-1	pETDuet-1 MCS1::SULT1A1$_{Rno}$-*cysPUWA*	Amp^r	This work
pCBJ364	pRSFDuet-1	pRSFDuet-1 MCS1::*cysZ*	Kan^r	This work
pCBJ365	pRSFDuet-1	pRSFDuet-1 MCS1::*cysPUWA*	Kan^r	This work
pCBJ368	pRSFDuet-1	pRSFDuet-1 MCS1::*cysP*$_{Bsu}$ MCS2::*cysDNCQ*	Kan^r	This work
pCBJ369	pRSFDuet-1	pRSFDuet-1 MCS1::*sbp-cysUWA* MCS2:*cysDNCQ*	Kan^r	This work
pCBJ372	pRSFDuet-1	pRSFDuet-1 MCS1::*cysP*$_{Bsu}$	Kan^r	This work
pCBJ373	pRSFDuet-1	pRSFDuet-1 MCS1::*sbp-cysUWA*	Kan^r	This work
pCBJ434	pRSFDuet-1	pRSFDuet-1 MCS2:*cysQ*	Kan^r	This work

[a] Kan^r, kanamycin resistance; Sp^r, spectinomycin resistance; Amp^r, ampicillin resistance.

1. Jensen, N. B. *et al.* EasyClone: Method for iterative chromosomal integration of multiple genes in Saccharomyces cerevisiae. *FEMS Yeast Res.* **14**, 238–248 (2014).

2. Jendresen, C. B. *et al.* Highly Active and Specific Tyrosine Ammonia-Lyases from Diverse Origins Enable Enhanced Production of Aromatic Compounds in Bacteria and Saccharomyces cerevisiae. *Appl. Environ. Microbiol.* **81**, 4458–4476 (2015).
