# Supplementary material for: Production of zosteric acid and other sulfated phenolic biochemicals in microbial cell factories
Source: Nat Commun. 2019 Sep 6;10:4071. doi: 10.1038/s41467-019-12022-x (PMC6731281; doi:10.1038/s41467-019-12022-x)
Supplement: Supplementary file 8 — Supplementary Data 4 [file 41467_2019_12022_MOESM8_ESM.pdf]

Supplementary Data 4

Production of zosteric acid and other sulfated phenolic biochemicals in microbial cell factories

Jendresen *et al*

Supplementary Data 4: Strains

**Strain**	**Construct/Genotype**	**Description**	**Source or reference**
Saccharomyces cerevisiae			
CEN.PK102-5B	MAT**a** *ura3-52 his3Δ1 leu2-3/112* $MAL2\text{-}8^{c}$ *SUC2*		Verena Siewers (Chalmers University)
CBJ973	CEN.PK102-5B/pCBJ282	PPGK1-$TAL_{Fjo, Sc}$	[1]
CBJ974	CEN.PK102-5B/pCBJ283	PTEF1-$SULT1A1_{Rno}$	This work
CBJ975	CEN.PK102-5B/pCBJ284	PTEF1-$SULT1A1_{Rno, Ec}$	This work
CBJ976	CEN.PK102-5B/pCBJ285	PTEF1-$SULT1A1_{Rno, Ec,}$ PPGK1-TAL_{Ses}	This work
CBJ978	CEN.PK102-5B/pCBJ287	PTEF1-$SULT1A1_{Rno, Ec,}$ PPGK1-TAL_{Fjo}	This work
CBJ980	CEN.PK102-5B/pCBJ289	PTEF1-$SULT1A1_{Rno, Ec,}$ PPGK1-$TAL_{Fjo, Sc}$	This work
CBJ981	CEN.PK102-5B/pCfB132	pCfB132	[1]
Escherichia coli			
NEB5-alpha	*fhuA2* D(*argF-lacZ)U169 phoA glnV44* f80D*(lacZ)*M15 *gyrA96 recA1 relA1 endA1 thi-1 hsdR17*		New England Biolabs
KRX	[F′, *traD36*, Δ*ompP*, *proA*$^{+}$*B*$^{+}$, *lacI*q, Δ(*lacZ*)M15] Δ*omp*T, *endA1*, *recA1*, *gyrA96* (Nal^{r}), *thi-1*, *hsdR17* (r_k^-, m_k^+), e14^{-} (McrA^{-}), *relA1*, *supE44*, Δ(*lac-proAB*), Δ(*rhaBAD*)::T7 RNA polymerase.		Promega
BL21(DE3)	F– *ompT hsdS*$_{B}$(r_B^- m_B^-) *gal dcm* (DE3)		Novagen, Life Technologies
CBJ893	NEB-alpha/pCBJ282	PPGK1-$TAL_{Fjo, Sc}$	[1]
CBJ894	NEB-alpha/pCBJ283	PTEF1-$SULT1A1_{Rno}$	This work
CBJ895	NEB-alpha/pCBJ284	PTEF1-$SULT1A1_{Rno, Ec}$	This work
CBJ896	NEB-alpha/pCBJ285	PTEF1-$SULT1A1_{Rno, Ec,}$ PPGK1-TAL_{Ses}	This work
CBJ898	NEB-alpha/pCBJ287	PTEF1-$SULT1A1_{Rno, Ec,}$ PPGK1-TAL_{Fjo}	This work
CBJ900	NEB-alpha/pCBJ289	PTEF1-$SULT1A1_{Rno, Ec,}$ PPGK1-$TAL_{Fjo, Sc}$	This work
CBJ1013	BL21(DE3)/pCBJ256+pCDFDuet-1	$SULT1A1_{Rno}$	This work
CBJ1014	BL21(DE3)/pCBJ256+pCBJ215	$SULT1A1_{Rno}$, TAL_{Rsp}	This work

CBJ1015	BL21(DE3)/pCBJ256+pCBJ297	SULT1A1$_{Rno}$, TAL$_{Rca}$	This work
CBJ1016	BL21(DE3)/pCBJ256+pCBJ228	SULT1A1$_{Rno}$, TAL$_{Fjo}$	This work
CBJ1041	BL21(DE3)/pCBJ256+pCBJ272	SULT1A1$_{Rno}$, CysDNCQ	This work
CBJ1053	BL21(DE3)/pCBJ256+pRSFDuet-1	SULT1A1$_{Rno}$	This work
CBJ1054	BL21(DE3)/pETDuet-1+pCBJ272	CysDNCQ	This work
CBJ1055	BL21(DE3)/pETDuet-1+pRSFDuet-1		This work
CBJ1091	KRX/pCBJ256	*R. norvegicus* SULT1A1	This work
CBJ1093	KRX/pCBJ337	*D. rerio* SULT1ST1	This work
CBJ1095	KRX/pCBJ339	*D. rerio* SULT6B1	This work
CBJ1136	KRX/pCBJ255	*H. sapiens* SULT1A1	This work
CBJ1138	KRX/pCBJ258	*D. melanogaster* dmST1	This work
CBJ1139	KRX/pCBJ259	*D. melanogaster* dmST1 - clone2	This work
CBJ1140	KRX/pCBJ260	*D. melanogaster* dmST3 - variant A	This work
CBJ1141	KRX/pCBJ261	*D. melanogaster* dmST4	This work
CBJ1142	KRX/pCBJ262	*E. caballus* SULT1A1	This work
CBJ1143	KRX/pCBJ263	*G. gallus domesticus* SULT1E1	This work
CBJ1144	KRX/pCBJ264	*C. lupus familiaris* SULT1A1	This work
CBJ1145	KRX/pCBJ265	*S. scrofa domesticus* SULT1A1	This work
CBJ1146	KRX/pCBJ320	*G. gallus domesticus* SULT1B1 - clone1	This work
CBJ1147	KRX/pCBJ321	*G. gallus domesticus* SULT1B1 - clone2	This work
CBJ1148	KRX/pCBJ322	*G. gallus domesticus* SULT1B1 - clone3	This work
CBJ1149	KRX/pCBJ323	*G. gallus domesticus* SULT1B1-predicted - clone1	This work
CBJ1150	KRX/pCBJ324	*G. gallus domesticus* SULT1B1-predicted - clone2	This work
CBJ1151	KRX/pCBJ325	*G. gallus domesticus* SULT1C1	This work
CBJ1153	KRX/pCBJ327	*G. gallus domesticus* SULT2B1-predicted	This work
CBJ1155	KRX/pCBJ329	*R. norvegicus* SULT1A1 (codon-opt)	This work
CBJ1156	KRX/pCBJ336	*C. elegans* SSU-1	This work
CBJ1157	KRX/pCBJ338	*D. rerio* SULT4A1	This work
CBJ1158	KRX/pCBJ340	*A. thaliana* AtSOT12	This work
CBJ1159	KRX/pCBJ341	*Streptomyces* Cpz8	This work
CBJ1160	KRX/pCBJ342	*Streptomyces* LipE	This work
CBJ1161	KRX/pCBJ343	*S. punctatus* DAOM BR117 SPPG_07427	This work
CBJ1162	KRX/pCBJ344	*H. ochraceum* DSM 14365 Hoch_6098	This work
CBJ1163	KRX/pCBJ345	*R. radiotolerans* RradSPS_0172	This work
CBJ1164	KRX/pCBJ346	*Zostera marina* KMZ76263.1	This work
CBJ1165	KRX/pCBJ347	*Zostera marina* KMZ64288.1	This work
CBJ1166	KRX/pCBJ348	*Zostera marina* KMZ76264.1	This work
CBJ1167	KRX/pCBJ349	*Zostera marina* KMZ59959.1	This work
CBJ1168	KRX/pCBJ350	*Zostera marina* KMZ74024.1	This work
CBJ1169	KRX/pCBJ351	*Zostera marina* KMZ64284.1	This work
CBJ1170	KRX/pCBJ352	*Zostera marina* KMZ72298.1	This work
CBJ1171	KRX/pCBJ353	*Zostera marina* KMZ72292.1	This work
CBJ1172	KRX/pCBJ354	*Zostera marina* KMZ72296.1	This work

CBJ1173	KRX/pCBJ355	*Zostera marina* KMZ76265.1	This work
CBJ1174	KRX/pCBJ356	*Zostera marina* KMZ73756.1	This work
CBJ1175	KRX/pCBJ357	*Zostera marina* KMZ69186.1	This work
CBJ1177	KRX/pETDuet-1		This work
CBJ1242	BL21(DE3)/pCBJ332+pCBJ272	$SULT1A1_{Rno}$, CysZ, CysDNCQ	This work
CBJ1243	BL21(DE3)/pCBJ333+pCBJ256	$SULT1A1_{Rno}$, CysZ, CysDNCQ	This work
CBJ1244	BL21(DE3)/pCBJ334+pCBJ256	$SULT1A1_{Rno}$, CysPUWA, CysDNCQ	This work
CBJ1246	BL21(DE3)/pCBJ228+pETDuet-1	TAL_{Fjo}	This work
CBJ1254	BL21(DE3)/pCBJ256+pCBJ271	$SULT1A1_{Rno}$, CysDNC	This work
CBJ1255	BL21(DE3)/pCBJ361+pCBJ272	$SULT1A1_{Rno}$, CysPUWA, CysDNCQ	This work
CBJ1256	BL21(DE3)/pCBJ256+pCBJ364	$SULT1A1_{Rno}$, CysZ	This work
CBJ1257	BL21(DE3)/pCBJ256+pCBJ365	$SULT1A1_{Rno}$, CysPUWA,	This work
CBJ1258	BL21(DE3)/pCBJ256+pCBJ368	$SULT1A1_{Rno}$, $CysP_{Bsu}$, CysDNCQ	This work
CBJ1259	BL21(DE3)/pCBJ256+pCBJ369	$SULT1A1_{Rno}$, Sbp-CysUWA, CysDNCQ	This work
CBJ1260	BL21(DE3)/pCBJ256+pCBJ372	$SULT1A1_{Rno}$, $CysP_{Bsu}$	This work
CBJ1261	BL21(DE3)/pCBJ256+pCBJ373	$SULT1A1_{Rno}$, Sbp-CysUWA	This work
CBJ1262	BL21(DE3)/pCBJ228+pCBJ256+pRSFDuet-1	$SULT1A1_{Rno}$, TAL_{Fjo}	This work
CBJ1264	BL21(DE3)/pCBJ228+pCBJ256+pCBJ272	$SULT1A1_{Rno}$, TAL_{Fjo}, CysDNCQ	This work
CBJ1266	BL21(DE3)/pCBJ228+pCBJ256+pCBJ334	$SULT1A1_{Rno}$, TAL_{Fjo}, CysPUWA, CysDNCQ	This work
CBJ1292	BL21(DE3)/pCBJ228+pCBJ256+pCBJ368	$SULT1A1_{Rno}$, TAL_{Fjo}, $CysP_{Bsu}$, CysDNCQ	This work
CBJ1389	BL21(DE3)/pCBJ256+pCBJ434	$SULT1A1_{Rno}$, CysQ	This work

1. Jendresen, C. B. *et al.* Highly Active and Specific Tyrosine Ammonia-Lyases from Diverse Origins Enable Enhanced Production of Aromatic Compounds in Bacteria and Saccharomyces cerevisiae. *Appl. Environ. Microbiol.* **81**, 4458–4476 (2015).
